# Bovine pulp extracellular matrix hydrogel for regenerative endodontic applications: in vitro characterization and in vivo analysis in a necrotic tooth model

**DOI:** 10.1186/s13005-024-00460-y

**Published:** 2024-10-22

**Authors:** Hisham Elnawam, Abdelrahman Thabet, Ahmed Mobarak, Nesma Mohamed Khalil, Amr Abdallah, Samir Nouh, Rania Elbackly

**Affiliations:** 1 Conservative Dentistry Department, Faculty of Dentistry, Alexandria University, Alexandria, Egypt; 2 Tissue Engineering Laboratories, Faculty of Dentistry, Alexandria University, Alexandria, Egypt; 3 Faculty of Dentistry, Champollion street, Azarita, Alexandria Egypt; 4 Oral Biology Department, Faculty of Dentistry, Alexandria University, Alexandria, Egypt; 5 Surgery Department, Faculty of Veterinary Medicine, Alexandria University, Alexandria, Egypt

**Keywords:** Biomimetic scaffolds, Cell-homing, Dental pulp extracellular matrix hydrogel, Dentin-pulp regeneration, Regenerative endodontics

## Abstract

**Background:**

Regenerative endodontic procedures (REPs) offer the promise of restoring vitality and function to a previously necrotic and infected tooth. However, the nature of regenerated tissues following REPs remains unpredictable and uncontrollable. Decellularized extracellular matrix scaffolds have gained recent attention as scaffolds for regenerative endodontics.

**Objectives:**

Preparation and characterization of a bovine dental pulp-derived extracellular matrix (P-ECM) hydrogel for regenerative endodontic applications. Biocompatibility and regenerative capacity of the prepared scaffold were evaluated in vivo in a canine animal model.

**Methods:**

Fifteen freshly extracted bovine molar teeth were used to prepare P-ECM hydrogels following approval of the institutional review board of the faculty of dentistry, Alexandria University. Decellularization and lyophilization of the extracted pulp tissues, DNA quantification and histological examination of decellularized P-ECM were done. P-ECM hydrogel was prepared by digestion of decellularized pulps. Prepared scaffolds were evaluated for protein content and release as well as release of VEGF, bFGF, TGF-β1 and BMP2 using ELISA. Rabbit dental pulp stem cells’ (rDPSCs) viability in response to P-ECM hydrogels was performed. Finally, proof-of-concept of the regenerative capacity of P-ECM scaffolds was assessed in an infected mature canine tooth model following REPs versus blood clot (BC), injectable platelet-rich fibrin (i-PRF) or hyaluronic acid (HA). Statistical analysis was done using independent t test, the Friedman test and chi-square tests (p value ≤ 0.05).

**Results:**

DNA was found to be below the cut-off point (50 ng/mg tissue). Histological evaluation revealed absence of nuclei, retention of glycosaminoglycans (GAGs) and collagen content, respectively. P-ECM hydrogel had a total protein content of (493.12 µg/µl) and protein release was detected up to 14 days. P-ECM hydrogel also retained VEGF, bFGF, TGF-β1 and BMP2. P-ECM hydrogel maintained the viability of rDPSCs as compared to cells cultured under control conditions. P-ECM hydrogel triggered more organized tissues compared to BC, i-PRF and HA when used in REPs for necrotic mature teeth in dogs. Periapical inflammation was significantly less in HA and P-ECM groups compared to blood-derived scaffolds.

**Conclusion:**

Bovine dental pulp-derived extracellular matrix (P-ECM) hydrogel scaffold retained its bioactive properties and demonstrated a promising potential in regenerative endodontic procedures compared to conventional blood-derived scaffolds.

**Supplementary Information:**

The online version contains supplementary material available at 10.1186/s13005-024-00460-y.

## Background

Over the past decade, regenerative endodontic strategies have enhanced the survival and improved the prognosis of hundreds of immature necrotic permanent teeth allowing their longer retention and offering a better quality of life for patients [1].These regenerative strategies have also been recently suggested as an alternative treatment modality for mature necrotic teeth as well. This has been elucidated by the successful short term clinical and radiographic results of multiple clinical trials as well as systematic reviews with meta-analyses. Despite this promising approach, several drawbacks and limitations have been shown warranting further investigation. These include tooth discoloration, progressive intracanal calcification, and lack of true tissue regeneration. The incidence of failed cases due to persistent residual infection has also been published. These challenges have fuelled the search for novel biomaterials and approaches that could trigger more tissue-specific repair/regeneration. Furthermore, the choice of intracanal medicament has been shown to have crucial effects on the outcome of regenerative endodontic procedures (REPs). While triple antibiotic paste has been advocated by several studies, calcium hydroxide remains the medicament of choice as reported by several studies. In a multinational survey from 13 countries, it was found that the majority of practitioners reported using calcium hydroxide as the intracanal medicament of choice for REPs [2, 3].

Ideally, the goal of REPs is to regenerate vital native-like tissues to reestablish the functions of the dentin-pulp complex through nociception and activation of the immune system. This would promote better tooth longevity and maintain the tooth’s ability to defend itself against noxious stimuli/pathogens^(4)^. While “regeneration” implies complete anatomical and functional restoration of the original tissue, the term ‘Endodontic regeneration’ or ‘regenerative endodontic procedures’ more broadly refers to the concept of ‘restoring’ or ‘regenerating’ tissues in place of those that were necrotic thereby providing the possibility of ‘revitalization’. This implies the re-establishment of a vital milieu capable of restoring nociception and immune competency [4]. Therefore, current research focuses on reaching more “predictable” outcomes regarding the success rates of regenerative endodontic procedures (REPs), as well as the nature of the regenerated tissues which should ideally mimic their native counterparts.

Regenerative endodontic procedures have been executed through two main approaches, the cell-based approach which involves the transplantation of stem cells from an exogenous source, and the cell-homing approach which aims to harness the body’s own response acting on the endogenous cells and factors bypassing the need for exogenous delivery [5]. Cell-based strategies have demonstrated appealing results, however, they do not represent a feasible and cost-effective approach for applied clinical translation of regenerative endodontic procedures [6]. This has led to the boost in cell-homing strategies which rely on upregulating wound healing to create the optimal regenerative niche leading to tissue regeneration [7, 8]. Indeed, regenerative endodontic strategies in their conventional definition may be viewed as cell-homing strategies in which the recruitment of host endogenous cells fulfils the triad of tissue engineering by facilitating de novo tissue regeneration and relying on the blood clot formed as an organic 3D scaffold, aided by activated platelets and organic dentin matrix to release signaling molecules [9].

Recently, the use of dental pulp-derived extracellular matrix (P-ECM) scaffolds has been highlighted as potential naturally derived scaffolds for dentin-pulp tissue engineering [10, 11]. These consist of a collagenous network, growth factors and glycoproteins that can provide structural and bio-chemical support for surrounding cells. They represent a promising cell-homing approach that has the ability to enhance cell recruitment, proliferation, release of signaling molecules from the surrounding microenvironment as well as stimulating cell differentiation [12]. Careful decellularization and crosslinking protocols to prepare extracellular matrix scaffolds derived from xenogeneic sources can produce biocompatible and cost-effective scaffolds for dental pulp regeneration [12–14]. Moreover, the use of a bovine P-ECM would have major advantages including the abundance of its source, utilizing this waste material and avoiding the ethical issues related to human-derived tissues [12, 15].

Although several studies have demonstrated the successful preparation and characterization of P-ECM scaffolds, none have demonstrated the potential of an injectable P-ECM hydrogel as a scaffold for REPs in infected necrotic mature teeth in a large animal model [11–13]. Therefore, in the current study, a decellularized bovine pulp-derived extracellular matrix (P-ECM) injectable hydrogel was prepared, characterized, and investigated for its bioactive potential. Moreover, the influence of using the P-ECM hydrogel scaffolds was evaluated in a dog orthotopic model, in comparison to the blood clot (BC), injectable-platelet-rich fibrin (i-PRF) or hyaluronic acid (HA) as scaffolds.

## Materials and methods

### Study design (Fig. 1)

The study was conducted in two phases; phase I included the preparation and characterization of bovine P-ECM hydrogel and phase II included the in vivo evaluation of the regenerative potential of the prepared P-ECM hydrogel. The Animal Research: Reporting of In Vivo Experiments (ARRIVE) guidelines [16] were followed during conduction of this research (Supplementary file 1). The in vitro experiments were done in triplicates. Since the in vivo part was a preliminary proof-of-concept experimental study, it was done on two dogs (16 teeth constituting 32 root canals). Each dog was operated on, in three stages, to fulfil the experimental procedure starting from establishing the necrotic tooth model, followed by canal disinfection and finally scaffold implantation and coronal seal. The graphical representation of the study design is provided as supplementary Fig. 1 (supplementary file 2).

**Fig. 1:**
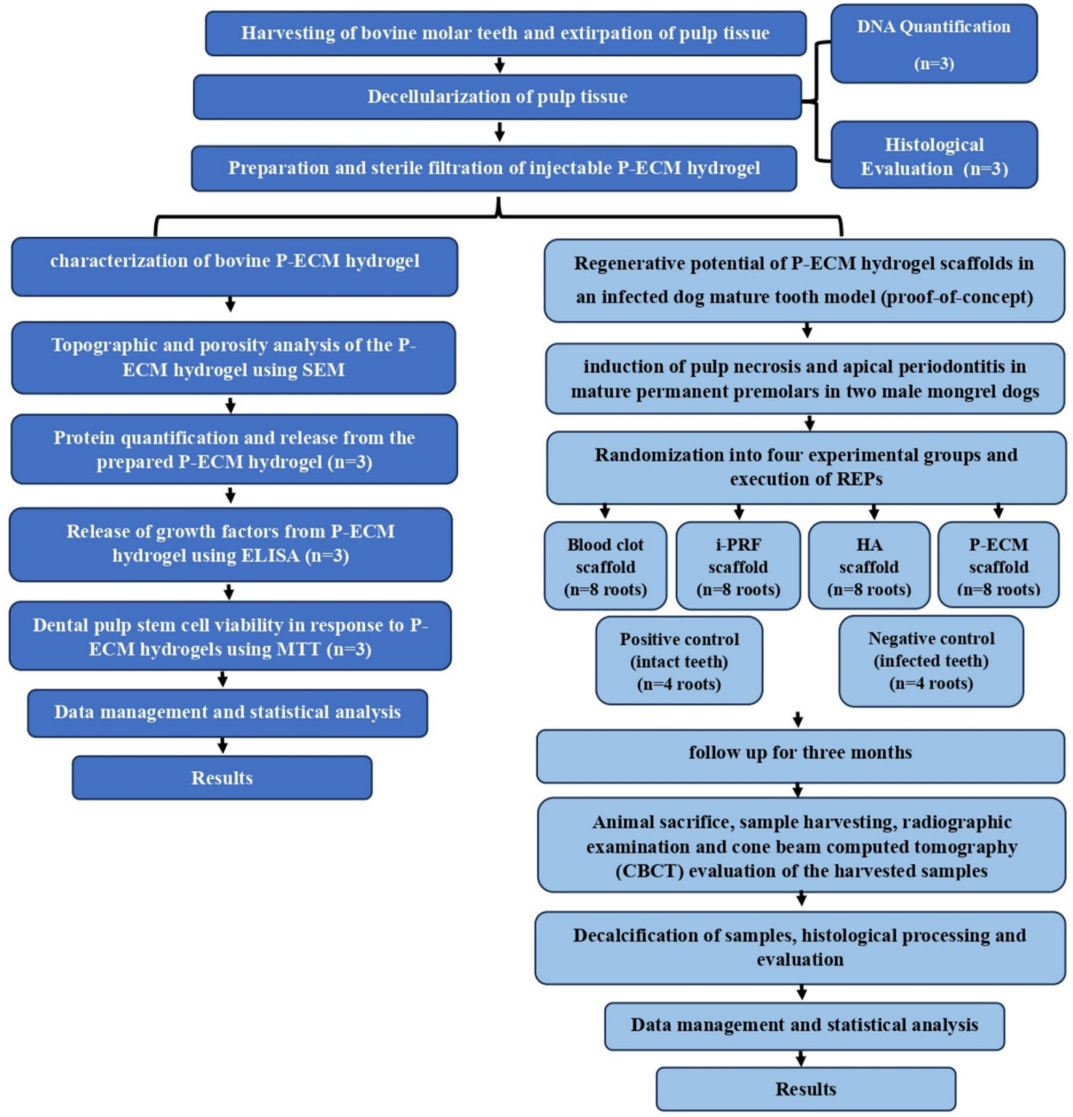
Flowchart of the experimental procedures (**CBCT**, cone beam computed tomography; **ELISA**, enzyme-linked immunosorbent assay; **HA**, hyaluronic acid; **i-PRF**, injectable platelet rich fibrin, **MTT**, 3-(4,5-dimethylthiazol-2-yl)-2,5-diphenyl-2 H-tetrazolium bromide assay; **P-ECM**, pulp extracellular matrix; **SEM**, scanning electron microscopy)

### Study setting

All in vitro and in vivo tests were done by one experienced investigator at the Tissue Engineering Laboratories, Faculty of Dentistry, Alexandria University, Egypt and following approval of the institutional review board of the Faculty of Dentistry, Alexandria University, Egypt (IRB No. 00010556- IORG No. 0008839) (0264-06/2021).

### Phase I: Preparation and characterization of bovine P-ECM hydrogel

#### Preparation and decellularization of bovine pulp tissue

**Fig. 2:**
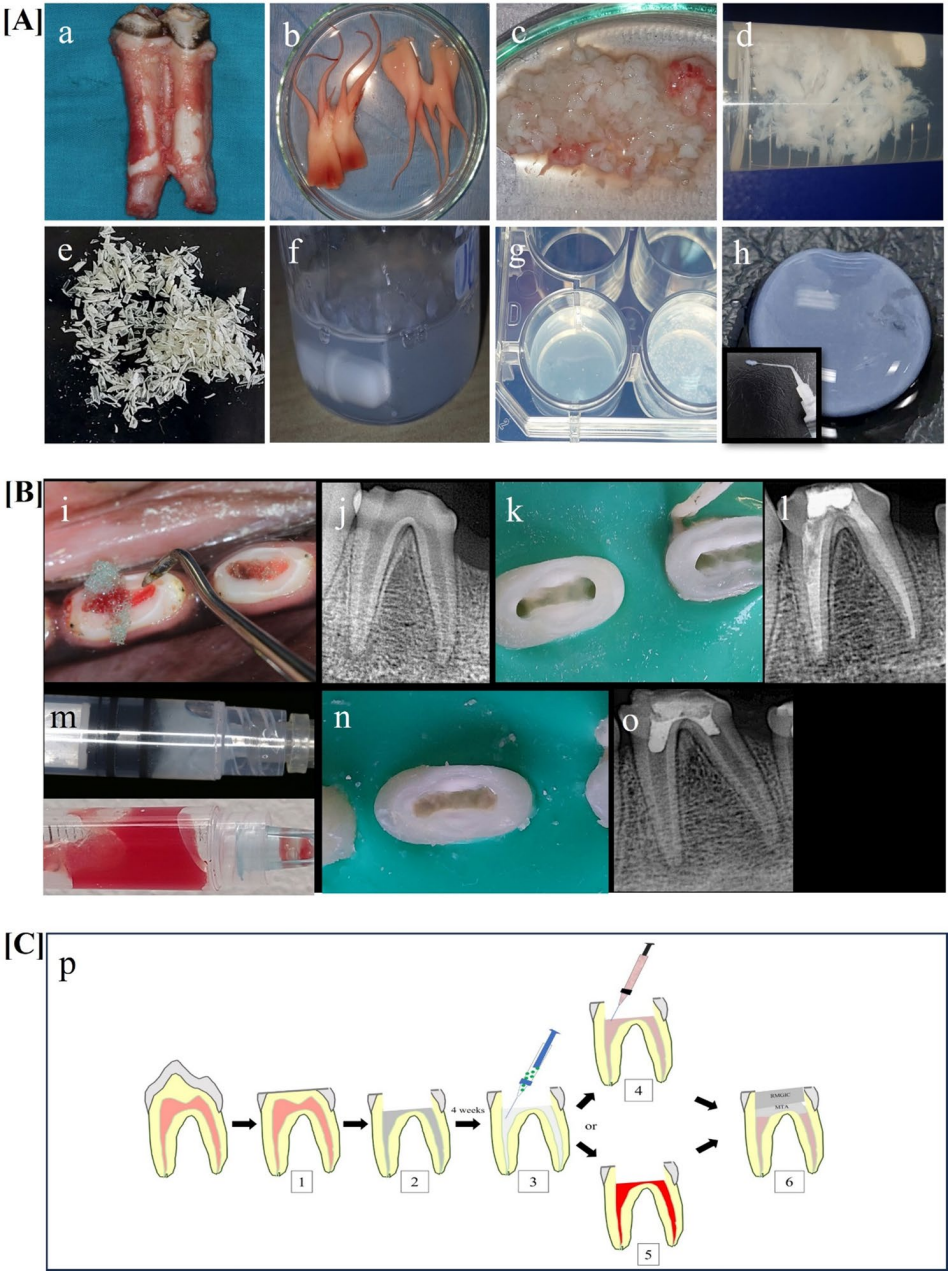
Methodology done in the current study. Upper panel (**A**): tissue harvesting and decellularization and P-ECM hydrogel preparation. (**a**) bovine mandibular molar teeth were immediately extracted; **(b&c)** bovine pulp tissues were extirpated, cut into equal pieces and decellularized using trypsin/EDTA, Dnase I and subsequent washes with PBS and deionized water; **(d)** decellularized pulp tissue ready for lyophilization; **(e)** lyophilized P-ECM after grinding; **(f)** digestion of tissues in pepsin/HCl for 24 h on stirrer; **(g)** pre-gel solution loaded in 24-well plate; **(h)** hydrogel with a concentration of 3 mg/mL after gelation; (Insert) in (**h**) showing injectability of P-ECM hydrogel. **Middle panel (B): regenerative endodontic procedures in a canine dog infected tooth model.** (**i**) access cavity preparation, extirpation of pulp tissue and induction of apical periodontitis; (**j**) follow up digital radiograph to confirm the formation of periapical lesions; (**k**&**l**) disinfection protocol and intracanal medicament placement for 2 weeks and IRM placement; (**m**) injection of hydrogels (P-ECM or HA) or injection of i-PRF; (**n**) MTA plug placement; (**o**) post operative digital radiograph confirming the coronal seal by MTA plug and resin modified GIC. **Lower panel (C): diagrammatic illustration showing** (1) The modification of the occlusal table of double-rooted premolars, (2) Access cavity preparation, extirpation of pulp tissue and induction of apical periodontitis, (3) Disinfection protocol and intracanal medicament placement for 2 weeks followed by either, (4) Injection of hydrogels (P-ECM, HA or i-PRF) or, (5) Induction of blood clot to fill the canal, (6) Coronal seal by MTA plug and resin modified glass ionomer cement

Mandibular molar teeth (*n* = 15) were obtained from ten 350–400 kg cows (mean age 1.5-2-year-old males) that were slaughtered for food production purposes at a veterinary-controlled slaughterhouse. Teeth were washed using sterile Phosphate Buffered Saline (PBS; Lonza, USA), and maintained on ice during transport. A hammer and chisel were used to crack the teeth open while sterile barbed broaches (Mani, Japan) and tweezers were used to extirpate the pulp tissues. The harvested tissue was washed with phosphate-buffered saline (PBS) at room temperature (RT), weighed, and stored at − 40 °C until decellularization (Fig. 2a, b &c). For decellularization, a modified protocol from Bakhtiar et al. [17] was used. The pulp tissue was minced by scalpel blades (approx. 5 × 5 mm) then decellularized; the ratio of tissue to liquid volume was 1:20 for best tissue wash and penetration. The minced pulp tissue was washed 4 times with deionized water for 1 h then subjected to treatment with 0.05% Trypsin, 0.02% EDTA (Biowest, France) at 37 °C for 24 h. The tissue was then washed with sterile PBS for 3 h, treated with 1% Triton X-100 (Loba Chemie, India) for 1 h and then washed again with PBS for 24 h at 4 ºC. The tissue was treated with nuclease solution DNase (Enzynomics, South Korea) for 1 h at 37 °C washed with sterile PBS for 24 h (at 4º C) and finally washed with deionized water for another 24 h (at 4º C). The tissues were weighed and stored at − 40 °C until further processing. After verification of the decellularization process, the tissues were lyophilized for 12 h and ground into small fragments (Fig. 2 upper panel; c, d &e).

#### DNA quantification and evaluation of presence of nuclei, collagen and glycosaminoglycans

DNA was extracted using the Genetix DNAsure tissue mini kit (Genetix Biotech LTD, India) according to manufacturer’s instructions. DNA was quantified using nanodrop 2000 spectrophotometers (ThermoSientific, USA) and QuantiFluor dsDNA Dye (Promega, USA) using the Luminescence Fluoro-meter plate reader (FLUOstar Omega, USA) following manufacturer’s instructions.

For histological analysis, formalin-fixed lyophilized native and decellularized pulps were dehydrated in a graded ethanol series (Merck Millipore, Germany), embedded in paraffin, and dissected into 5 μm sections. The tissue slides were stained with hematoxylin and eosin (H&E), Masson’s Trichrome (MT) or Alcian blue (AB) and observed under a light microscope (BX41; Olympus, Japan).

#### Preparation and characterization of injectable P-ECM hydrogel

Fifteen mg of finely milled freeze-dried P-ECM were digested in a solution of (pepsin (Sigma, USA)/ HCl) for 24 h at room temperature, under constant stirring, to obtain a final concentration of 3 mg/mL^(12)^. While samples were on stirrer and in cold water baths, 1 M sodium hydroxide (NaOH) was added dropwise until pH 7 was achieved and PBS 10x was added to each sample (with a ratio of 1:9). For sterilization of the hydrogels, pre-gel solution was immediately filtered using 0.45 μm polyvinylidene fluoride (PVDF) syringe filter (Labfil, China) [18, 19] then stored at -40º C until further use (Fig. 2 upper panel; f, g & h).

#### Topographic and porosity analysis of the P-ECM hydrogel

The pre-gel solutions (500 µl) of P-ECM hydrogels were placed in 24-well plate and incubated at 37 °C for 90 min to form hydrogel. P-ECM hydrogels were fixated in 2.5% glutaraldehyde (Electron Microscopy Sciences, England) for 24 h. The hydrogels were washed thrice with PBS 1x for 30 min at 4 °C. Then dehydrated in graded ethanol series. Hydrogels were then rinsed with 3 additional washes of absolute ethanol for 30 min/each, then they were air dried overnight at room temperature in fume hood. Samples were fixed on stubs and gold-sputter coated with 4.5 nm thickness. Surface topology and structure of hydrogels were viewed under scanning electron microscope (SEM; JEOL, Japan) at 2500x, 5000x and 10000x magnification. Image analysis was done using image analysis software (Image J 1.41, NIH, USA) [20] to view pore size and fibres diameter at magnification 10000x.

#### Protein quantification and release from P-ECM hydrogel

The total protein content for hydrogels was quantified using the bicinchoninic acid (BCA) assay kit (Biobasic, Canada) following manufacturer’s instructions and measured at 562 nm on micro-plate reader (AccuReader, Taiwan). P-ECM pre-gel solutions were prepared and recorded as a baseline concentration. Three hundred µl of pre-gel were loaded in cryopreservation tubes (in triplicates) and incubated at 37 °C overnight until complete evaporation of supernatant solution. 300 µl of PBS 1x was added to all tubes. Tubes were briefly spun, and supernatants were transferred to new Eppendorf tubes. The time intervals were 0-hour, 1 day, 5 days and 14 days. Protein content in each supernatant as well as in the negative control was detected using the BCA assay kit at 562 nm on micro-plate reader (AccuReader, Taiwan).

#### Release of growth factors from P-ECM hydrogel

Growth factors involved in angiogenesis (vascular endothelial growth factor [VEGF]), cell proliferation and collagen production (basic fibroblast growth factor [bFGF]), fibroblast function (transforming growth factor β1 [TGF-β1]) and regulation of mineralization (Bone morphogenetic protein 2 [BMP2]), were quantified in P-ECM pre-gel and P-ECM supernatants. Concentrations of VEGF, bFGF, TGF-β1 and BMP2 were assayed using ELISA kits according to the manufacturer’s instructions (bovine VEGF Immunoassay and bovine bFGF Immunoassay; BT labs (China), bovine TGF-β1 Immunoassay; Cloud-Clone (USA) and human BMP2 Immunoassay; R&D Systems (USA)).

#### Dental pulp stem cell viability in response to P-ECM hydrogels

Rabbit dental pulp stem cells (rDPSCs) were isolated from two New Zealand white rabbits (males, 3–4 weeks old, and with an average weight of 2–3 kg). 3-(4,5-dimethylthiazol-2-yl)-2,5-diphenyl-2 H-tetrazolium bromide (MTT) (Serva, Germany) cell viability assay was done using previously characterized rDPSCs [21]. Passage 3–5 cells were cultured in DMEM supplemented with 1% penicillin-streptomycin and 10% fetal bovine serum (FBS) and plated in uncoated or P-ECM-coated 24-well plates at a seeding density of 30,000 cells/well. Coating of the plates was done using 200 µl of pre-gel solution/well. Plates for incubated for 12 h at 37 ºC. The seeded plates (coated with P-ECM or uncoated) were incubated at 37 ºC in CO_2_ incubator for 24 h. The plates were evaluated using Inverted phase contrast microscope (TE2000; Nikon, Japan) then washed twice using PBS 1x. At each time interval (24 h and 3 days), MTT (0.25 mg/mL) was added then absolute ethanol was added. Ethanol was collected in 96 well plates and measured at 570 and 650 nm (Tecan, USA) (*n* = 3, in triplicate).

### Phase II: Regenerative potential of P-ECM hydrogel scaffolds in an infected dog mature tooth model (proof-of-concept)

#### Animal model

Two healthy male mongrel dogs aged 1.5–2 years with a complete set of permanent dentition were included for this study. Each dog was subjected to a full physical examination by an expert veterinarian (S.N., Professor of veterinary surgery, with more than 25 years of experience), to exclude any diseased dogs and kept under observation in separate cages (2 m x 2.5 m x 4 m). Dogs were housed under proper conditions of ventilation, nutrition, cleaning and a 12-hour light/dark cycle. They were given two meals of soft food daily and clean water. The standard diet regimen was refilled daily throughout the experimental period. Post-operative pain management and monitoring of animal behaviour including any changes in dietary habits or physical activities was checked daily by an expert veterinarian and the main investigator, to check for any clinical signs of distress. Following each intervention, Meloxicam (Delta Pharma Co., Egypt) was given at a daily dose of 1 mg/kg for 7–10 days postoperatively for pain management.

#### Randomization of samples and allocation concealment

In each dog, 8 premolars were used with a sum of 16 teeth constituting 32 root canals. Teeth were randomly divided, using computer generated random numbers, into four experimental groups (*n* = 8 roots/ group). Teeth were allocated to one of the following groups: blood clot (BC) scaffold, injectable platelet rich fibrin (i-PRF) scaffold, hyaluronic acid (HA) scaffold or pulp extracellular matrix (P-ECM) scaffold. In each dog, four other roots were used as negative control (necrotic teeth with no treatment) and another four roots were used as positive control (intact teeth).

#### Establishment of necrotic mature tooth canine model ([11, 22])

Animals were sedated by I/M injection of 2% Xylazine HCL (ADWIA Co., Egypt) at a dose of 1 mg/kg by weight followed by I/M injection of Ketamine HCL (Rotex medica, Germany) at a dose of 5–10 mg/kg body weight. Inhalation anaesthesia was done by Isoflurane (ACDIMA international trading, Egypt) to maintain sedation. Local anaesthesia was then administered via Articaine 4% (Septodont, France). Preoperative radiographs were done using a digital X-ray sensor (Eighteeth, China). For induction of apical periodontitis, endodontic access cavities were prepared without isolation and pulps were disrupted using #15 and #20 K-files (Mani, Japan). Then a collected plaque suspension was injected into the cavities. Sterile sponges and cotton pellets were packed into access cavities and the root canals were left exposed to the oral cavity. After 4 weeks, apical periodontitis was radiographically confirmed.

#### Disinfection and regenerative endodontic procedures ([11, 22]) (Fig. 2 middle and lower panels [B] and [C])

All experimental procedures were performed under general and local anaesthesia as previously described. Magnifying loupes (Dr. Kim, USA) with a minimum of 4x magnification were used. Under rubber dam isolation, working length was radiographically confirmed followed by establishment of glide path starting with K file #10 and #15 (Mani, Japan). Cleaning and shaping were done up to apical size of 0.3 mm using X3 files (Protaper Next, Dentsply Sirona, USA) and each canal was irrigated with 20 mL of 3% sodium hypochlorite, followed by 20 mL of sterile saline; then dried with sterile paper points. Calcium hydroxide intracanal medicament (Ultradent, USA) was placed and access openings were sealed with Intermediate Restorative Material (IRM) (Dentsply Sirona, USA). Two weeks later, under the same general anaesthesia and aseptic techniques, the experimental teeth were re-entered, the temporary cement was removed, and the canals were irrigated with 20 mL of sterile saline, followed by 20 mL of 17% EDTA (Prevest Denpro, Jammu, India) followed by a final rinse with saline and drying of canals using sterile paper points.

For the blood clot (BC) group, bleeding was provoked to fill the whole canal up to the cemento-enamel junction and left to clot for 10 min. For i-PRF group, apical patency was ensured then i-PRF was injected into the canals up to the level of cemento-enamel junction. For the preparation of i-PRF; 10 mL of whole blood from the median cubital vein was drawn and divided equally into two 15-mL plastic tubes without anticoagulant reagent. Blood was centrifuged at 800 rpm for 3 min using Hettich EBA 20 centrifuge (Merck Pty. Ltd., Germany). After centrifugation, the upper liquid layer (i-PRF) was collected as close as possible to the layer of red cells [23]. For the HA group and P-ECM group, apical patency was ensured then the hydrogels were injected into the canals using a back-filling approach up to the level of cementoenamel junction to avoid unnecessary extruded material. Excess material was then removed using sterile cotton pellets. To ensure complete stability of the hydrogel, any excess moisture was absorbed using sterile gauze and the hydrogel was left in place for a minimum of 15 min before placement of the coronal MTA plug material.

For all experimental groups, mineral trioxide aggregate (MTA) (Cerkamed, Poland) plug was packed in the coronal 3 mm and radiographically confirmed followed by light cured resin modified glass ionomer cement (RMGIC) (SDI limited, Australia) to obtain coronal seal according to the guidelines of the American Association of Endodontists, 2021 [24]. No further coronal restoration was placed due to the short duration of the study. Post-operative radiographs were taken.

#### Animal sacrifice, sample harvesting, radiographic examination and cone beam computed tomography (CBCT) evaluation of the harvested samples

After three months following the regenerative endodontic procedures, animals were sacrificed with an overdose intravenous injection of 20 mL of 5% Thiopental sodium. En-bloc samples of the teeth embedded in the jaw bones were harvested. The specimens were then fixed in 10% neutral buffered formalin for 1 week. Post-mortem digital radiographic and CBCT evaluations were done by two blinded investigators. Radiographic findings such as the presence or absence of intracanal hard tissue, periapical radiolucency or root resorption were tabulated.

#### Decalcification protocol for the harvested samples

En-bloc samples of the teeth embedded in the jaw bones were rinsed in distilled water for 24 h to remove residual formalin and decalcified in 14% EDTA solution at pH 8 (Oxford Lab Chem, India) on an orbital shaker for 4–6 months.

#### Histological evaluation

Decalcified specimens were dehydrated in a series of ethyl alcohol and encased in paraffin blocks. Micro-sections were cut mesiodistally at a thickness of 5 μm. Slides were stained with hematoxylin and eosin dye. On selected sections, Masson’s trichrome staining was used for further analysis. The samples were evaluated, under a light microscope, by two blinded examiners (H.E. and N.M.K). Study examiners underwent thorough training on the histological outcome criteria. Any discrepancies or disagreements in scores interpretation were addressed and resolved through consensus. Following this training, histological sections were independently evaluated by the two examiners on two separate occasions, one week apart. Inter-examiner reliability was assessed, with values ranging from 0.84 (95% CI [0.73, 0.94]) to 0.88 (95% CI [0.81, 0.93]) indicating acceptable agreement. The analyses were done on 4 serial sections from every sample of each group. The prepared histological slides labels were covered with scotch tape for blinding purposes during histological examination and analysis.

Criteria for identification were as follows: presence of intracanal fibrous connective tissue in the canal; presence of mineralized tissues resembling either dentin, cementum, or bone in the canal; periapical inflammation represented by presence of inflammatory cell infiltrates in the periapical tissue; root resorption represented by presence of osteoclastic /odontoclastic activity on the root apex. Periapical inflammation was categorized as none, mild (0), moderate (1), or severe (2) based on the number of inflammatory cells within the high-power field (0 cells/field, ˂50 cells/field, > 50 cells/ field, or packed field, respectively). The level of new tissue in-growth was also scored [22, 25, 26]. Gram staining was used to detect the presence of any residual bacteria in the dentinal tubules of control samples and different experimental groups following REPs. The stained slides were examined using the oil immersion technique under a light microscope with a high magnification power (1000X) [27, 28].

### Statistical analysis

Normality of all measured outcomes were tested using Shapiro Wilk test and Q-Q plots. Normal distribution was confirmed for DNA quantification, protein release, fiber diameter, and porosity while other outcomes were not normally distributed. The parametric independent t test was used to compare between decellularized and native pulp. The non-parametric Friedman test followed by a post hoc test with Bonferroni correction was used to assess changes across time points regarding protein release, VEGF, bFGF TGF-β1 and BMP2. Qualitative data for histological and radiographic analysis of dogs’ samples were described using number and percent and data were analyzed using non-parametric Chi Square test. All tests were two tailed and the significance level was set at p value ≤ 0.05. Data were analyzed using IBM SPSS for Windows version 23, Armonk, NY, USA. The graphical presentation was done using GraphPad Prism version 10.0.0 for Windows, GraphPad Software, USA.

## Results

### Removal of cells and nuclear material and preservation of native architecture following decellularization of bovine dental pulp tissue (Fig. 3A)

Histological examination of native and decellularized bovine pulp samples revealed the absence of nuclei in decellularized pulp and retention of collagen and glycosaminoglycans (GAGs), respectively. DNA content of decellularized P-ECM was found to be 32 ± 2.4 ng/mg tissue using Nanodrop spectrophotometer and 21.18 ± 1.8 ng/mg tissue using Quantifluor fluorescent dye. While for native pulp tissue it was found to be 182.4 ± 3.5 ng/mg tissue using Nanodrop spectrophotometer and 350.7 ± 5.6 ng/mg tissue using Quantifluor fluorescent dye which was significantly different between native and P-ECM (*p* ≤ 0.0001) (supplementary file 3, Tables 1 and 2).

**Fig. 3:**
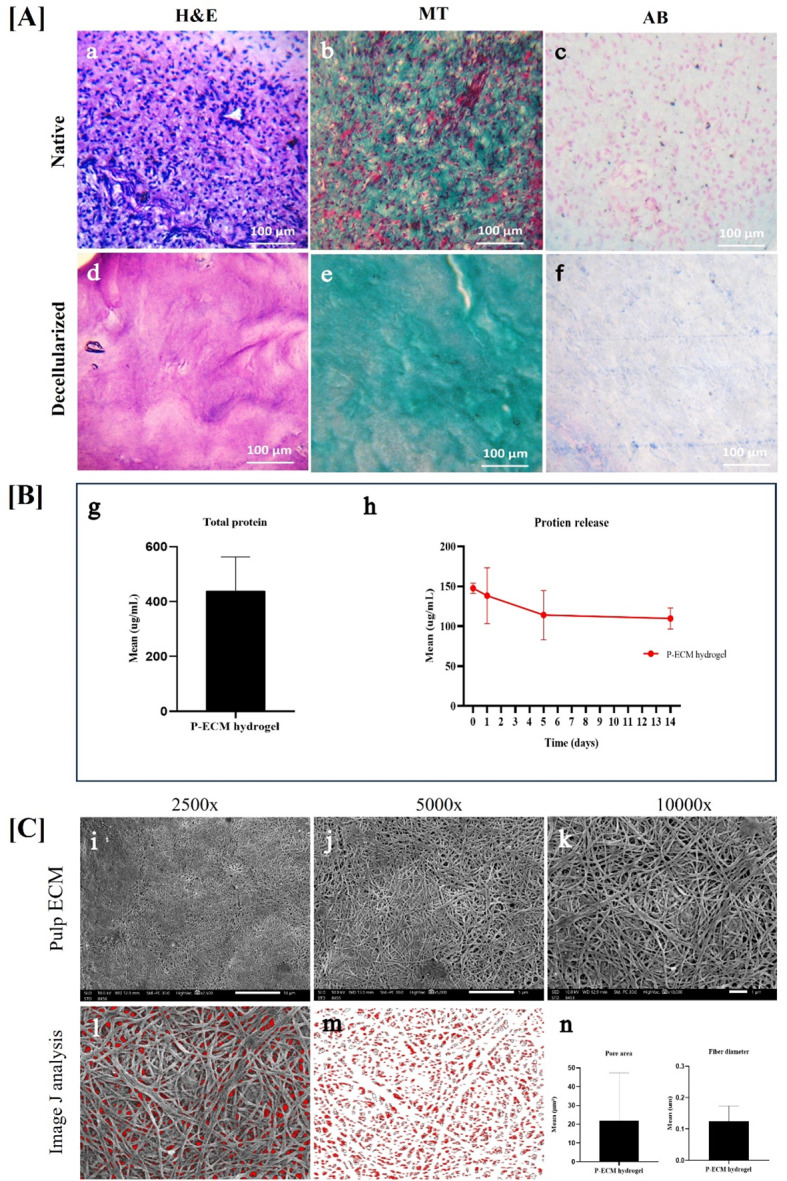
Results of histological characterization, protein content, protein release and scanning electron microscopic analysis. Upper panel [**A**]: showing the histological analysis of decellularized P-ECM in comparison to native pulp tissue (**a**) native pulp at 10x magnification stained with H&E “nuclei stained purple”; **(b)** native pulp at 10x magnification stained with Masson’s Trichrome (MT) stain “nuclei stained dark red”; (**c)** native pulp at 10x magnification stained with Alcian Blue (AB) stain “nuclei stained pink”; **(d**,** e and f)** showing decellularized P-ECM at magnification “10x” stained with **H**&**E**, MT and AB, respectively where absence of nuclei is evident in all decellularized sections. Collagen bundles (stained blue by MT stain) and tissue architecture were preserved following decellularization. GAGs content (stained faint blue by Ab stain) is evident in both native and decellularized tissues. **Middle panel [B]: protein quantification and pattern of release g)**: bar graph showing mean protein content in P-ECM hydrogel; (**h**) line graph showing the release pattern of proteins in P-ECM hydrogel where it shows the highest release at 0 day and gradually decreases over a period of 14 days. **Lower panel [C]: scanning electron microscopic SEM images of P-ECM (i**,** j&k)** surface structure of P-ECM at 2500x, 5000x and 10000x magnifications, respectively. **(l**,** m&n)** ImageJ analysis for fibers’ diameter and mean pore area of P-ECM hydrogel

### Protein quantification and release patterns in P-ECM hydrogels (Fig. 3B)

Total protein content was found to be 439 ± 123.4 µg/mL in P-ECM hydrogels. Protein release was detected at time 0 showing a burst release 147.56 ± 6.29 µg/mL followed by a decline at 24 h 138.21 ± 35.03 µg/mL which was then followed by a steady decrease at day 5 114.04 ± 30.75 µg/mL and almost reaching a plateau at day 14 109.79 ± 13.12 µg/mL. There were no statistically significant differences between timepoints (supplementary file 3, Tables 3 and 4).

### Surface topography and pore size of the prepared P-ECM hydrogel (Fig. 3C)

SEM image analysis revealed an average fiber diameter of 0.13 ± 0.05 μm and a mean pore area of 21.82 ± 25.48 µm^2^.

### The retention and release pattern of growth factors in P-ECM hydrogel

**Fig. 4:**
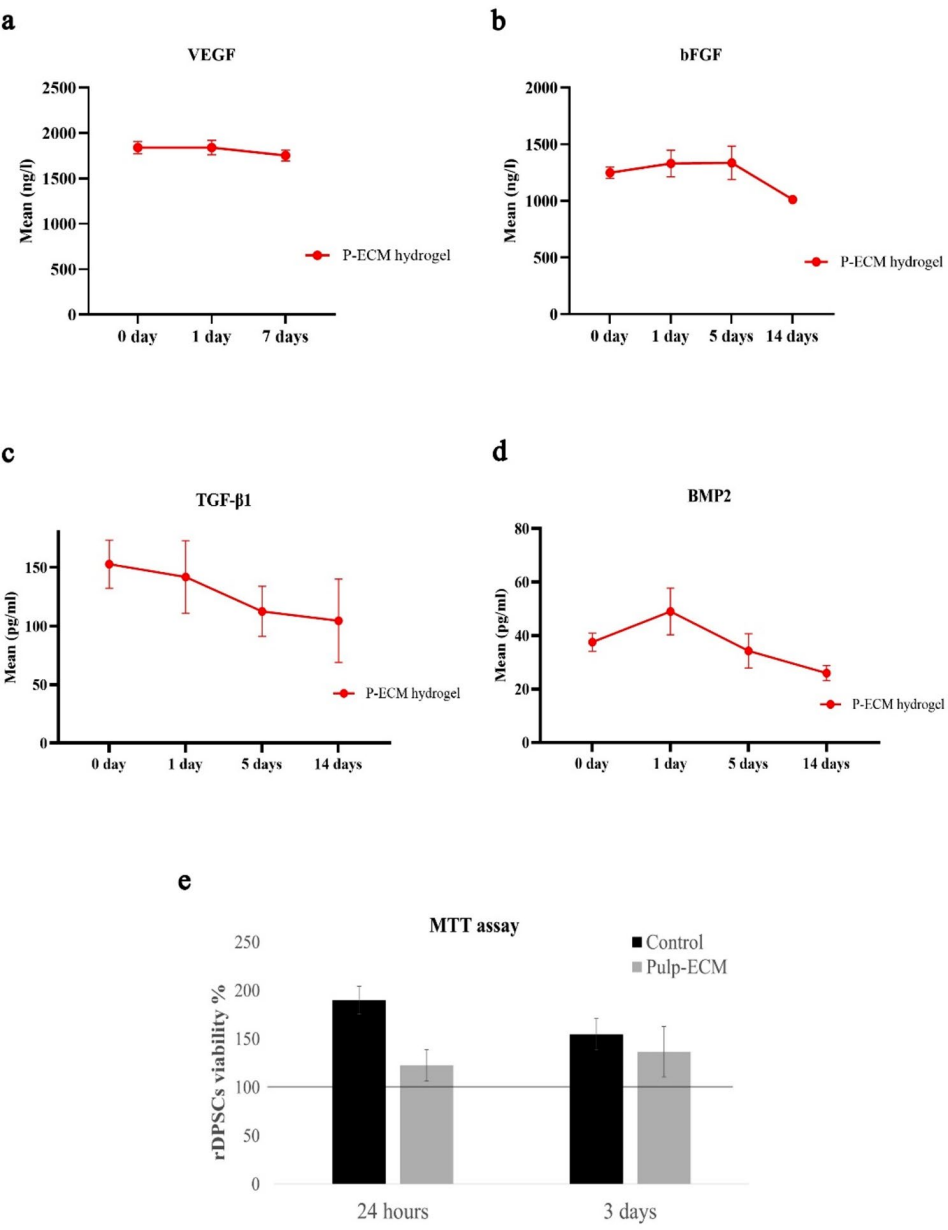
Diagrammatic representation of results of growth factors’ quantification in P-ECM hydrogels by ELISA and viability assay in response to rDPSCs. **(a)** line graph showing the release curve of VEGF in ng/L in P-ECM hydrogel at 0 days, 1 day, and 7 days; **(b)** line graph showing the release curve of bFGF in ng/L in P-ECM at 0 days, 1 day, 5 days and 14 days; **(c)** line graph showing the release curve of TGF-β1 in pg/mL in P-ECM at 0 days, 1 day, 5 days and 14 days; **(d)** line graph showing the release curve of BMP2 in pg/mL in P-ECM hydrogel at 0 days, 1 day, 5 days, and 14 days; **(e)** bar graph showing rDPSCs viability % after 24 h and 3 days in response to P-ECM. Viability % was calculated by normalizing the absorbance values to those of 30,000 rDPSCs measured at baseline

Regarding vascular endothelial growth factor (VEGF), the mean VEGF content in P-ECM pre-gel was 776.80 ± 18.67ng/L. As for its pattern of release, it showed almost sustained release up to 7 days where it was detected at 0 h to be 1839.90 ± 68.31 ng/L, 1841.30 ± 79.05 ng/L at day 1 and 1753.00 ± 59.40 ng/L at day 7 (Fig. 4a). Basic fibroblast growth factor (bFGF) mean content in P-ECM pre-gel was 1416.50 ± 231.12 ng/L. It showed nearly sustained release from 0 h 1249.05 ± 50.68 ng/L, 1330.14 ± 117.57 ng/L at day 1 and 1336.49 ± 147.98 ng/L at day 5. This was followed by declined release until day 14 1012.24 ± 5.65 ng/L (Fig. 4b). Regarding transforming growth factor β1, mean TGF-β1 content in P-ECM pre-gel was 158.33 ± 36.76 pg/mL. It showed a slowly decreasing release rate, where it showed a burst release at 0 h 152.76 ± 20.54 pg/mL then started to gradually decrease 141.79 ± 30.90 pg/mL at day 1, 112.49 ± 21.43 pg/mL at day 5 then 104.46 ± 35.58 pg/mL at day 14 (Fig. 4c). Finally, the mean bone morphogenetic protein 2 content in P-ECM hydrogel was 305.06 ± 10.84 pg/mL. It was detected at 0 h to be 37.56 ± 3.44 pg/mL followed by increased release at day 1 49.03 ± 8.76 pg/mL then it started to decrease to be 34.30 ± 6.39 pg/mL at day 5 and 25.98 ± 2.82 pg/mL at day 14 (Fig. 4d). These differences were not statistically significant (supplementary file 3, Tables 5, 6, 7 and 8).

### Influence of P-ECM hydrogel on rDPSCs viability (Fig. 4e)

No cell cytotoxicity was observed in the experimental group compared to the control group. Although cells proliferated for both groups after 24 h, cell proliferation in response to the P-ECM coated plates was less than for the control conditions. After 3 days, while cells appeared to start to plateau in the control condition, they appeared to regain their viability and proliferation capacity at day 3.

### Radiographic and histological outcomes of regenerative endodontic procedures using P-ECM hydrogels versus BC, i-PRF or HA (Figs. 5, 6, 7 and 8)

There were no unexpected outcomes following the experimental interventions, including no changes in behaviour or eating patterns of the dogs. Following induction of apical periodontitis, radiographically, there were clear periapical lesions at the apices of all experimental teeth, indicating formation of chronic apical periodontitis which was more evident in negative control (infected) teeth. Positive control (intact) teeth showed an intact periodontal ligament and lamina dura in periapical radiographs. All teeth retrieved for analysis had intact coronal seal.

**Fig. 5:**
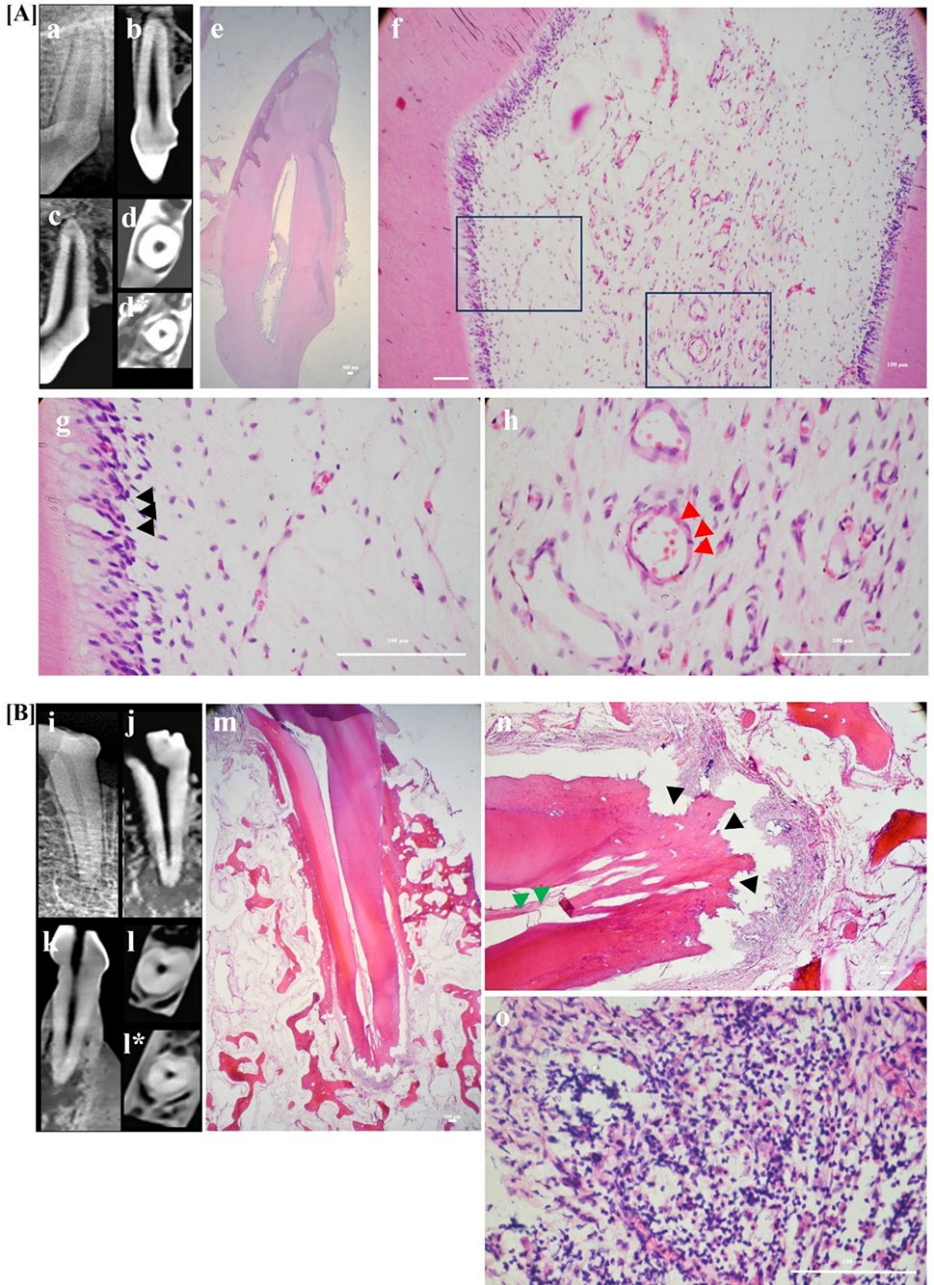
Upper panel [**A**]: Representative sample for positive control group showing CBCT analysis and photomicrographs of dog’s positive control (intact tooth) sample (**a**) Digital radiograph showing intact tooth. **(b)** Coronal view of CBCT image showing pulp space with no calcific tissues and normal periapical area. (**c)** Sagittal view of CBCT image showing positive control tooth with normal periapical area. (d&d*) Horizontal cuts of CBCT image showing middle and apical thirds of the root, respectively. Notice the absence of calcific tissues in normal canal space. (**e)** Photomicrograph showing normal periapical area **(f)** Positive control sample showing layers of normal pulp tissue; odontoblastic layer, cell-free zone, cell -rich zone and core of the pulp. **(g&h)** Same sample at higher magnification showing odontoblastic layer “black arrows” and highly vascularized normal pulp tissue with numerous blood vessels “red arrows”. (**H**&**E**: **e**, **f, g**& **h**); (10x: **e**. 40x: **f**, 400x: **g** & **h**). Lower panel [**B**]: Representative sample for negative control group showing CBCT analysis and photomicrographs of dog’s negative control (infected tooth) sample (**i**) Digital radiograph showing infected tooth 3 months following the access opening. (**j**) Sagittal view of CBCT image showing negative control tooth with large periapical lesion. **(k)** Coronal view of CBCT image showing pulp space with no calcific tissues and large periapical lesion. (l&l*) Horizontal cuts of CBCT image showing middle and apical thirds of the root, respectively. Notice the absence of calcific tissues in the canal space. (**m)** Photomicrograph showing canal space with no de-novo tissue regeneration and signs of severe root resorption and periapical inflammation. **(n)** Degenerated pulp tissue “green arrow heads”, multiple resorptive lacunae “black arrow heads” and severe inflammation. **(o)** Numerous inflammatory cells at the periapical area. (**H&E: m, n &o**); (10x: **m**, 40x: **n** 400x: **o**)

**Fig. 6:**
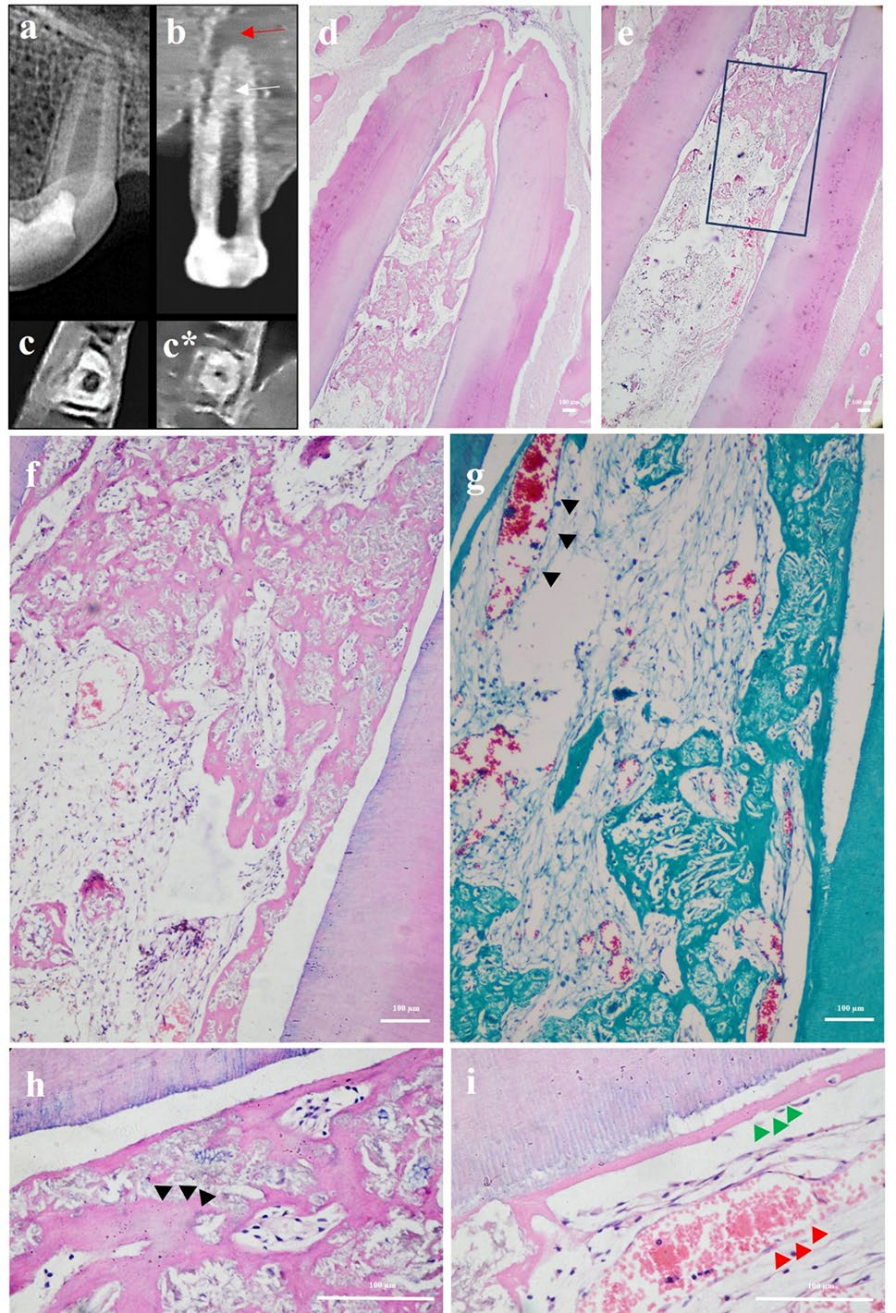
Representative sample for BC group showing CBCT analysis and photomicrographs of dog’s tooth treated with blood clot scaffold (**a**) Digital post operative radiograph showing a periapical lesion. **(b)** Coronal view of CBCT image showing pulp space with intracanal calcification “white arrow” and a periapical lesion “red arrow”. (c&c*) Horizontal cuts of CBCT image showing middle and apical thirds of the root, respectively. Notice the presence of intracanal calcific tissues. **(d&e)** Photomicrograph showing new tissue formation **(f&g)** Same sample at higher magnification showing highly vascularized connective tissue with numerous blood vessels “black arrow heads” and hard tissue formation. **(h&i)** Higher magnification of (**e**) showing bone-like tissue “black arrow heads”, cementum-like tissue “green arrow heads” and new blood vessels “red arrow heads”. (**H**&**E**: **d**, **e, f**, **h**& **i**); (MT: **g**); (40x: **d**&**e**); (100x: **f**&**g**); (400x: **h**&**i**)

**Fig. 7:**
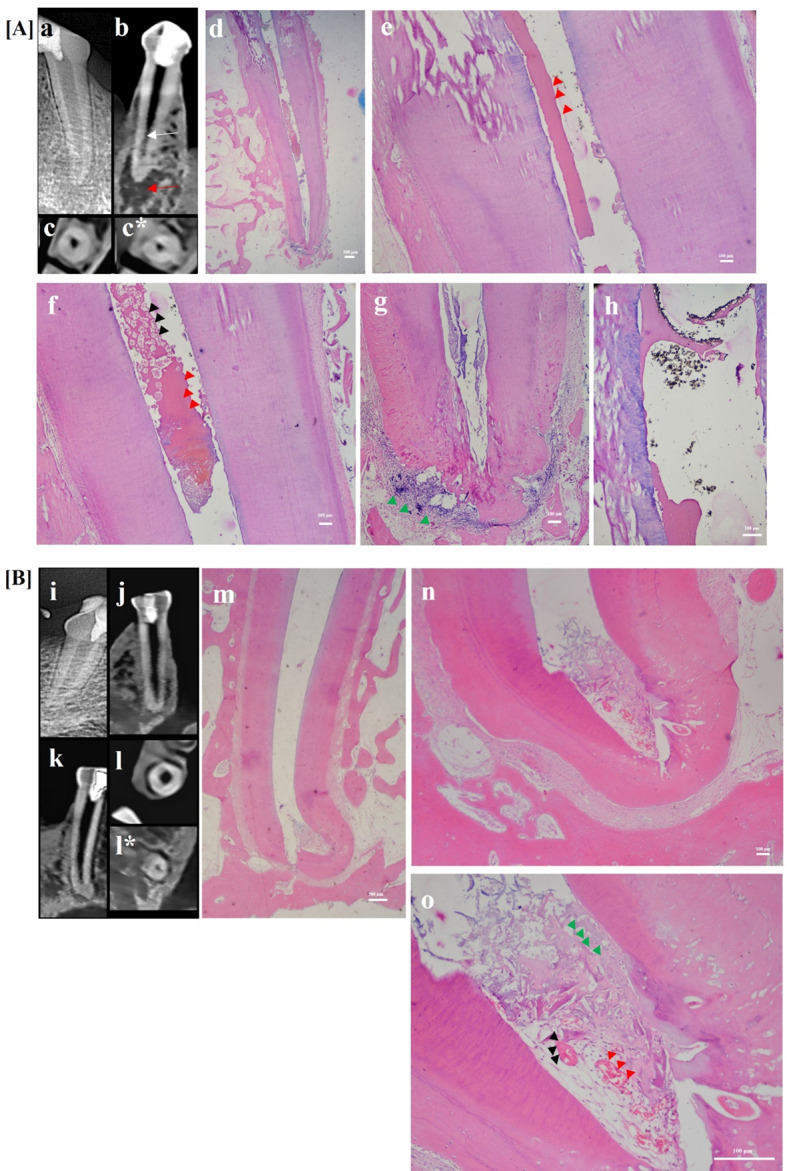
Upper panel [**A**]: showing representative sample for i-PRF group showing CBCT analysis and photomicrographs of dog’s tooth treated with blood clot scaffold (**a**) Digital post-operative radiograph showing a periapical lesion. **(b)** Horizontal view of CBCT image showing pulp space with intracanal calcific tissues “white arrow” and a periapical lesion “red arrow”. (c&c*) Horizontal cuts of CBCT image showing middle and apical thirds of the root, respectively, notice the presence of intracanal calcific tissues. **(d)** Photomicrograph showing periapical inflammation and mineralized tissue formation up to cervical third **(e&f)** Coronal and middle thirds, respectively, at higher magnification, showing bone-like “black arrow heads”, cementum-like “red arrow heads”. **(g)** Apical third showing periodontal-like tissue formation “green arrow heads”, notice the root resorption and inflammatory infiltrate at the periapical area. **(h)** Bridge formation under the MTA coronal plug (**H**&**E**: **d, e, f**, **g**&**h**); (10x: **d**; 40x: **e, f**&**g**; 100x: **h**). Lower panel [**B**]: showing representative sample for HA group showing CBCT analysis and photomicrographs of dog’s tooth treated with HA scaffold (**i**) Digital post-operative radiograph showing small periapical lesion. **(j)** Coronal view of CBCT image showing pulp space with minimal intracanal calcific tissues at apical third and a small sized periapical lesion. **(k)** Sagittal view of CBCT image showing pulp space with minimal intracanal calcific tissues at apical third and a small sized periapical lesion (**l**&l*) Horizontal cuts of CBCT image showing coronal and apical thirds of the root, respectively, notice the presence of intracanal calcific tissues only in apical third. **(m)** Photomicrograph showing periapical healing and tissue formation only at apical third **(n)** Showing periapical healing with no inflammatory cells and new tissue formation at the apical third of canal lumen. **(o)** Higher magnification of (**m**) showing cementum-like tissue “black arrow heads”, periodontal ligament PDL -like tissue “green arrow heads” and new blood vessels “red arrow heads”. (**H&E: m, n &o**); (10x: **m**; 40x: **n**; 100x: **o**)

**Fig. 8:**
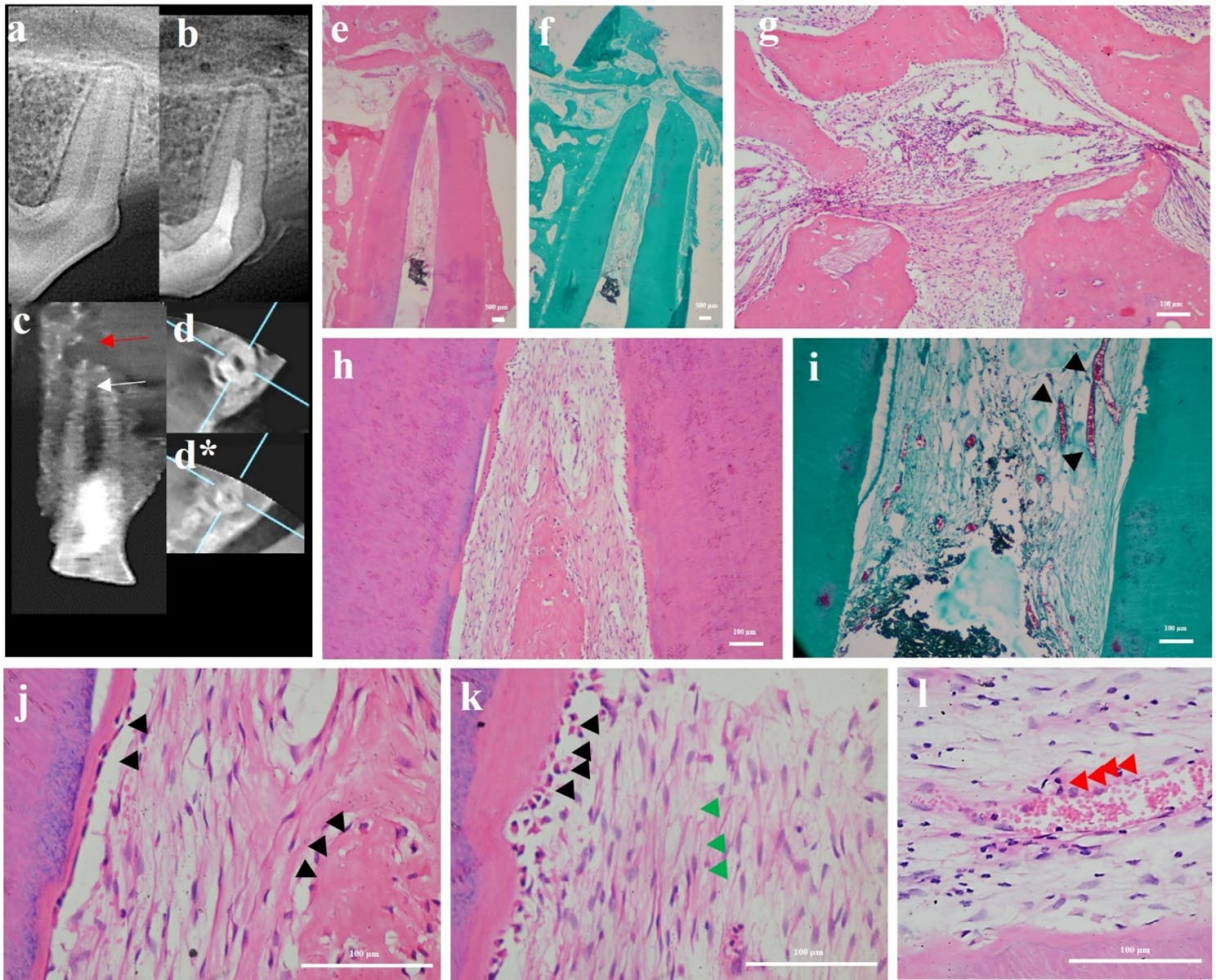
Representative sample for P-ECM group showing CBCT analysis and photomicrographs of dog’s tooth treated with P-ECM scaffold (**a**) Digital preoperative radiograph showing normal periapical condition. **(b)** Digital post-operative radiograph showing periapical lesion **(c)** Coronal view of CBCT image showing pulp space with minimal intracanal calcific tissues at apical third “white arrow” and a periapical lesion “red arrow”. (d&d*) Horizontal cuts of CBCT image showing middle and apical thirds of the root, respectively, Notice the presence of intracanal calcific tissues in the apical third. **(e&f)** Photomicrographs showing periapical healing and regenerated tissue up to the level of MTA coronal plug. **(g)** Apical third of canal showing periapical cementum healing and ingrowth of regenerated tissues **(h)** Middle third of canal showing vascularized soft tissue and mineralized tissue formation. **(i)** Same sample showing numerous blood vessels “black arrow heads”, dense connective tissue fibers (**j, k&l**) Higher magnification showing cementum-like tissue with cells lining the inner canal wall “black arrow heads”, dense connective tissue fibers “green arrow heads” and new blood vessels “red arrow heads”. (**H&E: e, g,h, j,k&l**); (MT: **f&i**); (10x: **e&f**; 100x: **g,h&i**; 400x: **j,k&l**)

Histological evaluation showed normal roots, pulp space, and periapical tissue in the positive control teeth (Fig. 5A; a-h). On the other hand, empty canals, root resorption and intense periapical inflammation were detected in the negative control teeth (Fig. 5B; i-o). Gram staining of negative control samples (infected tooth) showed numerous clusters of bacterial aggregations in different regions of dentinal tubules throughout the samples but particularly close to the canal lumen. On the other hand, dentinal tubules of the positive control samples (intact tooth) were found to be free from any bacterial contamination Additionally, none of the experimental groups showed any visible residual bacteria in their dentinal tubules as detected by gram staining (supplementary Fig. 2 in supplementary file 4).

Histologically, in the blood clot group (Fig. 6), majority of samples showed newly formed tissues that were highly vascularized connective tissue as well as intracanal cementum-like tissues filling the entire canal length in most samples. Bone-like tissue was also seen as well as periapical inflammation and root resorption in some samples. In the i-PRF group (Fig. 7A), less vascularized connective tissues as well as cementum-like and bone-like tissues were observed in most of the samples in which tissues were detected throughout the whole canal lumen. As for the HA group (Fig. 7B), there was intracanal hard tissue in half of the samples which was limited to the apical third. Mild periapical inflammation was seen and varying levels of root resorption. On the other hand, in the P-ECM group (Fig. 8), highly vascularized connective tissue was observed in most of the samples. It filled the canal up to the level of the MTA coronal plug in 62.5% of samples and up to the middle third in 12.5% of samples. Cementum-like tissue deposition with more organized cell arrangement was detected on internal canal walls of most samples with very mild inflammation and limited root resorption detected.

As for the comparison between the groups, intracanal hard tissue detected was significantly higher in the i-PRF and BC groups compared to the HA and P-ECM groups (*p* < 0.005). The detection of periapical radiolucency and root resorption were higher in the negative control group followed by the i-PRF group then the BC group while they were less evident in most of the samples in both the HA and P-ECM groups. Radiographic findings of control and experimental groups are shown in (Tables 1 and 2).

Histologically, in all groups, there was new tissue formation except in the negative control group. This de-novo tissue formation was more in the BC and i-PRF groups compared to all other groups but significantly more than the negative control (*p* < 0.042) (Figs. 6 and 7A). The level of new tissue in-growth was the highest for the i-PRF group followed by the BC then P-ECM groups and finally the HA group. This was significantly more than both the HA and negative control groups (*p* < 0.043), with no statistically significant difference between any of the other experimental groups. The i-PRF and BC groups showed the highest amount of mineralized tissue formation, followed by the P-ECM group and finally the HA group, with no statistically significant difference between the experimental groups.

The BC and i-PRF groups behaved similarly regarding the degree of periapical inflammation, showing significantly higher levels of inflammation compared to positive controls (*p* < 0.014). On the other hand, the HA and P-ECM groups had the least periapical inflammation as compared to negative control and i-PRF groups (*p* < 0.025) (Figs. 7B and 8). As for apical root resorption, it was the highest for the negative control group followed by the i-PRF group. Furthermore, it was significantly less in the BC, HA and P-ECM groups compared to the negative control (*p* < 0.044).

**Table 1:** Comparison of radiographic findings between different groups

Radiographic Findings		Positive (*n* = 4)	Negative (*n* = 4)	BC (*n* = 8)	i-PRF (*n* = 8)	HA (*n* = 8)	*P*-ECM (*n* = 8)
Intracanal hard tissue detected	No	4 (100%)	4 (100%)	1 (12.5%)	1 (12.5%)	8 (100%)	7 (87.5%)
	Yes	0 (0%)	0 (0%)	7 (87.5%)	7 (87.5%)	0 (0%)	1 (12.5%)
	P value	< 0.0001*					
	Post hoc test	BC vs. HA = 0.005*, BC vs. P-ECM = 0.033*, i-PRF vs. HA = 0.005*, i-PRF vs. P-ECM = 0.033*					
Periapical radiolucency	No	4 (100%)	0 (0%)	2 (25%)	1 (12.5%)	6 (75%)	7 (87.5%)
	Yes	0 (0%)	4 (100%)	6 (75%)	7 (87.5%)	2 (25%)	1 (12.5%)
	P value	0.001*					
	Post hoc test	i-PRF vs. P-ECM = 0.046*					
Root Resorption	No	4 (100%)	0 (0%)	5 (62.5%)	3 (37.5%)	7 (87.5%)	7 (87.5%)
	Yes	0 (0%)	4 (100%)	3 (37.5%)	5 (62.5%)	1 (12.5%)	1 (12.5%)
	P value	0.007*					
	Post hoc test	Negative vs. HA = 0.046*, Negative vs. P-ECM = 0.046*					

*Statistically significant at p value < 0.05

**Table 2:** Histological findings in experimental and control groups

Histological Findings		Positive (*n* = 4)	Negative (*n* = 4)	BC (*n* = 8)	i-PRF (*n* = 8)	HA (*n* = 8)	*P*-ECM (*n* = 8)
Intracanal tissue formation	No	-	4 (100%)	1 (12.5%)	1 (12.5%)	4 (50%)	2 (25%)
	Yes	-	0 (0%)	7 (87.5%)	7 (87.5%)	4 (50%)	6 (75%)
	P value	0.015*					
	Post hoc test	Negative vs. BC = 0.042*, Negative vs. i-PRF = 0.042*					
Level of new tissue in-growth	Score 0: No tissue	-	4 (100%)	1 (12.5%)	1 (12.5%)	4 (50%)	2 (25%)
	Score 1: Till apical third	-	0 (0%)	0 (0%)	0 (0%)	4 (50%)	0 (0%)
	Score 2: Till middle third	-	0 (0%)	1 (12.5%)	0 (0%)	0 (0%)	1 (12.5%)
	Score 3: Till cervical third	-	0 (0%)	6 (75%)	7 (87.5%)	0 (0%)	5 (62.5%)
	P value	0.001*					
	Post hoc test	Negative vs. i-PRF = 0.028*, i-PRF vs. HA = 0.043*					
Mineralized tissue formation	No	4 (100%)	4 (100%)	1 (12.5%)	1 (12.5%)	4 (50%)	2 (25%)
	Yes	0 (0%)	0 (0%)	7 (87.5%)	7 (87.5%)	4 (50%)	6 (75%)
	P value	0.003*					
	Post hoc test	NS					
Periapical inflammation	None	4 (100%)	0 (0%)	0 (0%)	0 (0%)	0 (0%)	0 (0%)
	Score 0: mild	0 (0%)	0 (0%)	2 (25%)	0 (0%)	8 (100%)	8 (100%)
	Score 1: moderate	0 (0%)	0 (0%)	6 (75%)	6 (75%)	0 (0%)	0 (0%)
	Score 2: Severe	0 (0%)	4 (100%)	0 (0%)	2 (25%)	0 (0%)	0 (0%)
	P value	< 0.0001*					
	Post hoc test	Positive vs. Negative < 0.0001*, Positive vs. BC = 0.014*, Positive vs. i-PRF < 0.0001*, Negative vs. HA = 0.005*, Negative vs. P-ECM = 0.005*, i-PRF vs. HA = 0.025*, i-PRF vs. P-ECM = 0.025*					
Root resorption	None	4 (100%)	0 (0%)	4 (50%)	0 (0%)	3 (37.5%)	4 (50%)
	Score 0: mild	0 (0%)	0 (0%)	3 (37.5%)	0 (0%)	3 (37.5%)	2 (25%)
	Score 1: moderate	0 (0%)	0 (0%)	1 (12.5%)	7 (87.5%)	2 (25%)	2 (25%)
	Score 2: Severe	0 (0%)	4 (100%)	0 (0%)	1 (12.5%)	0 (0%)	0 (0%)
	P value	< 0.0001*					
	Post hoc test	Positive vs. Negative = 0.002, Positive vs. i-PRF = 0.014*, Negative vs. BC = 0.011*, Negative vs. HA = 0.044*, Negative vs. P-ECM = 0.022*					

*Statistically significant at p value < 0.05

## Discussion

In regenerative endodontics, natural and synthetic biomaterials that can mimic dentin-pulp extracellular matrix to reproduce all its complex characteristics are still not available [29]. Decellularized extracellular matrix-derived scaffolds could offer a natural biomimetic alternative to conventional scaffolds for tissue engineering and regenerative medicine applications [30]. Extracellular matrix (ECM) scaffolds may be considered a viable option for tissue regeneration as they contain a cocktail of growth factors and bioactive molecules bypassing the need for specific growth factor delivery. This would not only support cell functions but would also dictate cell commitment and guide differentiation [31]. Although many ECM components such as hyaluronic acid [32] and collagen [33] have been tested clinically, decellularized ECM has not been yet accepted for clinical use in regenerative endodontics [30]. Indeed, hyaluronic acid (HA) hydrogels have been suggested as potential scaffolds for dentin/pulp regeneration owning to their biocompatibility and ability to allow preservation of extracellular spaces while at the same time preserving tissue mechanical integrity [5]. In addition, their biodegradation products have been shown to enhance angiogenesis besides the fact that hyaluronic acid is a major constituent of the extracellular matrix. Moreover, there are several FDA and European Commission approved formulations of HA for clinical use in a variety of applications [34]. However, their use for REPs has not been demonstrated in vivo in animal models.

In the present study, we report successful decellularization of bovine dental pulp which was then lyophilized, digested and prepared as an injectable hydrogel. Such a tissue specific injectable scaffold could be of particular clinical interest as it can be easily delivered into root canal spaces [35, 36]. Moreover, it can be administrated using minimally invasive techniques without requiring extensive canal preparation to place the scaffold. Additionally, following our decellularization protocol, P-ECM was found to retain its native architecture and structural integrity. This is in accordance with Bakhtiar et al. [12, 17] who reported that using Trypsin/EDTA-based decellularization protocol resulted in successful preparation of a three-dimensional macroporous pulp-derived scaffold with established physical, biological and regenerative characteristics. Recent studies by Matoug-Elwerfelli et al., reported favourable outcomes when using decellularized human dental pulp [37] or decellularized rat dental pulp [38]. Decellularized porcine dental pulp was also reported to have regenerative potential in the study of Alqahtani et al. [11]. However, a lack of sufficient tissue source for both human and rat dental pulps as well as the unavailability of porcine-derived tissues in some countries, may pose significant challenges for using such scaffolds in REPs. Therefore, a bovine source for decellularized dental pulp scaffolds could be more readily available [12]. Immunogenicity risk is a crucial aspect to be considered when selecting a scaffold for endodontic regeneration. In our study, a careful decellularization protocol was performed to overcome immunological limitations of xenogeneic materials which led to the preservation of ECM structural integrity while eliminating the cellular components. Residual DNA content was found to be below the cut-off point (50 ng of residual DNA per milligram of dry tissue) that would avoid triggering an immune response by the host [11, 12, 37]. Preserved collagenous and glycosaminoglycan structure of the dental pulp was confirmed by Masson’s Trichrome and Alcian blue staining, respectively. SEM analysis also confirmed the preservation of a collagenous network and surface porosity of the prepared hydrogel which is in accordance with Bakhtiar et al. [12].

The total protein content in the prepared hydrogel was quantified and it continued to be released over a period of two weeks. A two-week time period would coincide with the initial events involved in the wound healing cascade and the initiation of mineralization cascades [39–41]. Growth factors that are known to be present in native dental pulp and crucial for pulp regenerative processes including angiogenesis, cell proliferation and collagen production, fibroblast function and regulation of mineralization process were assessed [42–46]. VEGF, TGF-β1 and BMP2 have been shown to be essential for pulp regeneration and proved to have a synergistic effect with bFGF to enhance specific biological effects [47, 48]. Yadlapati et al. [49] reported that VEGF induces stem cell differentiation into endothelial cells and modulates tooth development and dentin formation. Basic fibroblast growth factor (bFGF) has been reported to contribute to the regulation of all aspects of tooth development, repair, and regeneration [43, 50] including mesenchymal stem cell migration, proliferation, and stemness maintenance, as well as dentin formation, angiogenesis, and neurogenesis [51, 52]. Maintaining the release of bFGF and TGF-β1 biological activity was examined by Mathieu et al. [53] by investigating their effect on dental pulp cell proliferation. They reported that their sustained release for up to 6 days increased cell proliferation which could enhance the early steps of dentin-pulp regeneration. Iohara et al. [54] reported that BMP2 directs pulp progenitor/stem cell differentiation into odontoblasts and results in dentin formation. Moreover, Yang et al. [55] reported that BMP2 is directly required for odontoblast differentiation and indirectly contributes to pulp vasculogenesis via VEGF-A production in odontoblasts. TGF-β1 has been shown to play a critical role in the cell-homing approach in regenerative endodontics. Its release from dentin following the use of EDTA has been shown to direct cell migration, proliferation and differentiation [56, 57]. In our study, we demonstrated that P-ECM hydrogel retained the forementioned growth factors as well as their release pattern over a period of 7 to 14 days. In this study both VEGF and bFGF showed almost sustained release during the first week. It was reported that dental pulp cells usually start proliferating from 3 to 7 days [58]. Therefore, a sustained release of factors related to cell proliferation, migration and angiogenesis during this period of time could be beneficial. It is worth mentioning that VEGF starts to elevate on the first day in wounded tissue healing and then significantly elevates till 7 days [59]. Nagaraja et al. [60] reported a different release pattern of VEGF in dental pulp where it was negligible at day 1 and 4 followed by a significant increase at day 7 (twice our findings). However, the inflammatory state of the extirpated pulp and the presence of VEGF receptors on pulp cells could contribute to the pattern of release in their study. Our results are in accordance to a study performed by Tran-Hung et al. [61] who reported a VEGF level of 769 pg/mL in supernates of cultured human intact pulp cells. Regarding bFGF concentrations it was in accordance to the those reported by Kim et al. [62] who stated that bFGF at the concentration 1 ng/mL increased the proliferation and differentiation potential of dental pulp cells. Despite methodological differences, TGF-*β*1 levels in our study were almost half the amount reported by Laurent et al. [63] where intact pulp cells were found to secrete TGF-*β*1 at a level of 374 pg/mL in an in vitro whole-tooth culture model. Although the levels of BMP2 detected were quite low compared to other studies that showed effective odontoblastic differentiation in response to BMP2, such high levels may not be required since these P-ECM scaffolds represent a reservoir of growth factors in their natural ratios required for de-novo dentin-pulp formation [54, 64]. 

Regarding the influence of P-ECM hydrogels on dental pulp stem cell viability, they showed no cytotoxic effects and maintained cell viability similarly to Bakhtiar et al. [12]. Although the there was an initial reduction of cell proliferation at 24 h in comparison to control conditions, the cells rapidly recovered after 3 days. While decellularization adequately removed any potential immunogenic cellular material, it has been shown that traces of materials and chemicals used during processing may result in some preliminary residual cytotoxicity [12]. On the other hand, the proliferation rate in the control group could have decreased due to the cells reaching a plateau (confluence) as a result of the high initial seeding density. A high seeding density was used since the objective of the experiment was to evaluate the effect of the P-ECM coating in the early post-seeding time (3 days) interval which would coincide with the early wound healing phase following injection of the gels in vivo. This result was in accordance with AbdulKhodir et al. who reported a similar observation [65].

As for in vivo endodontic regeneration, the animal model used in this study was chosen based on previous studies by Alqahtani et al. [11] and Alenazy et al. [66]. Large animals that have dental anatomy and tooth size comparable to humans allow for clinical simulation and evaluation of orthotopic pulp regeneration [67]. Therefore, in this study model we simulated a long-standing infection within the root canal space of mature permanent teeth eventually leading to periapical disease [67]. The disinfection protocol was the same in all our experimental groups. Intracanal medicament was used for 14 days following the recommendation of Holland et al. [68] who reported significant disinfection of dogs infected root canals when calcium hydroxide was placed for 14 days rather than 7 days.

Apical diameter enlargement was done up to 0.3 mm based on the findings of Laureys et al. [69] who reported that 0.3 mm was sufficient to allow tissue ingrowth in an animal model. In addition, Saoud et al. [70] and El-Kateb et al. [71] successfully treated mature maxillary anterior teeth with apical diameter sizes of 0.3 mm. Moreover, it has been shown that the average size of human cells ranges from 10 to 100 μm according to Estefan et al. [72]. Digital radiographs, micro-computed tomography (CBCT), and histological analysis were used to evaluate the teeth in this study. The use of digital radiographs might lead to underestimation of the lesion size and histological severity. Therefore, post-mortem CBCT scans provided images with higher resolution and allowed the assessment of teeth in a multiplanar view. In the present study, periapical lesions and root resorption were detected in most of the samples in the BC and i-PRF groups while they were not detected in most of the teeth treated by either P-ECM or HA. However, correlation between histology and radiology in dogs is poor [73]. The presence of a radiolucency does not always indicate apical periodontitis in the dog as dog teeth commonly show physiologic radiolucencies [74].

According to histological evaluation, there was evidence of periapical healing in all test groups, with varying degrees of residual periapical inflammation between different groups. The lowest degree of inflammation was noted in P-ECM and HA groups which could be due to the immunomodulatory potential of ECM components [7] as well as the antibacterial effects of P-ECM [75].

Newly formed mineralized tissues resembling cementum were consistently observed in the root canal space, attached to the inner dentin wall in the blood clot group. Moreover, newly formed connective tissues occupying the canal lumen were more like periodontal tissue rather than pulp tissue. This is comparable to the results reported by Alenazy et al. [66] who reported similar tissues to proliferate inside the canals of mature dog teeth following revascularization procedures. Regenerated tissues in the i-PRF group were similar to those in the BC group, however it appeared to trigger more hard tissue formation and less vascularized connective tissue which was in accordance to findings reported by Eldessoky et al. [76]. These findings could be attributed to the degree of periapical inflammation and the nature of recruited cells from surrounding periodontal ligament/alveolar bone and the absence of tissue-specific biological cues that would orchestrate their differentiation [77, 78].

In teeth treated with HA, most samples showed mild signs of periapical inflammation and root resorption. However, the amount of tissue in-growth was limited only to the apical portion of canal. This could be explained by the fact that while HA has anti-inflammatory properties, promotes stem cell differentiation and supports tissue integrity [79–82], its slow degradation rate might act as a physical barrier to tissue in-growth [83–85]. This was in accordance with the findings of Palma et al. [86] who demonstrated that after 13 weeks the scaffolds were maintained inside of the canal forming an obstacle for ingrowth of the apical tissues into the pulp space. Despite this, no traces of the scaffold were found in the samples of the current study. This could be due to the hydrogel form of the HA and its solubility during sample processing. However, using hyaluronic acid with lower molecular weight may allow tailoring of the hydrogels to allow faster biodegradation to better coincide with new tissue formation [87]. Well-organized highly vascular connective tissue was detected in canal lumens of P-ECM treated teeth with lesser amount of calcific tissue deposition. This might be due to the retained growth factors and ECM components within the scaffold that could influence the chemotaxis and commitment of surrounding stem cells [11, 88].

Regarding presence of residual bacteria, gram staining did not reveal observable bacterial contamination in any of the experimental groups. This was consistent with the findings reported by Gomes-Filho et al. [89] following REPs in necrotic mature dog teeth. According to Saoud et al. [70] the residual bacteria after the disinfection phase in REPs might have been eliminated by the immune defence mechanism of the newly formed intracanal tissues. Moreover, the absence of bacterial contamination in various samples might confirm the efficacy of the disinfection protocol suggested by the AAE, 2021 [24] which was followed in this study, together with the coronal bacterial-tight seal applied. It also might demonstrate that the concentrations used were effective in eliminating bacterial cells while maintaining viability of recruited stem/ progenitor cells and the capacity to generate new vital tissues [90].

Interestingly, although there were no clinical signs or symptoms of infection, several teeth from different groups still showed persistent periapical lesions at the end of the study. This could be related to the unique root canal anatomy of dogs’ teeth with multiple portals of exit and apical ramifications which may have contributed to the presence of residual bacterial antigens that may remain following disinfection [74]. Even after elimination of bacterial cells, residual bacterial antigens were found to affect the commitment of undifferentiated cells which makes the nature of regenerated tissues unpredictable [90, 91]. Moreover, in this experimentally infected tooth model, the severity and duration of induced apical periodontitis does not really recapitulate the real clinical situation. In most human clinical trials and case series, following-up the healing process of periapical lesions ranges between 12 and 24 months. A recent systematic review of clinical studies has reported that the majority of cases (63%) were detected after 12 months following initiation of treatment while 39% of failed REPs cases were identified after 24 months [92]. However, being unable to apply this in animal studies could result in underestimation of periapical healing. Other possible model-related factors include difference of microbiome in apical lesions and oral bacterial flora, difficulty in bleeding induction inside the canal and difference in healing pattern of apical inflammation [74].

It has been shown that the structure of dentin previously exposed to bacterial biofilms becomes altered which may negatively affect the migration, attachment, proliferation and differentiation of recruited stem and progenitor cells. Furthermore, previously infected dentin may sequester variable levels of growth factors as compared to natural healthy dentin [93]. This is in addition to the fact that it has been shown that the larger the periapical lesion and the longer the infection, the higher the virulence of microorganisms thereby creating a more challenging environment for tissue regeneration [94]. The more severe the infection is, the more is the residual inflammatory response and hence this has been shown to have detrimental effects on regenerated tissues following REPs in animal models [95].

In clinical cases with healing apical lesions, there is no histological evidence of new tissue formation inside root canals. Healing of apical periodontitis mainly depends on proper disinfection and elimination/reduction of bacterial load. The selection of a treatment modality such as REPs is still uncertain when treating a mature necrotic tooth with apical periodontitis until more reliable outcomes are available [96]. On the contrary, several studies have indicated that revitalization procedures have shown promising outcomes for such teeth demonstrating resolution of signs and symptoms, periapical healing, new hard tissue formation, and in many cases restored sensibility [71, 97]. Nevertheless, implementation of this approach for mature teeth is still an area of debate due to the inconsistency of outcomes and lack of robust comparative research and long-term follow-up. These outcomes are also greatly influenced by the scaffold type used in these revitalization procedures, a fact which propelled the execution of the current study [4]. Therefore, it is important to correlate between what is placed inside the canal and the direct effects it has on canal microenvironment as well as on the immune response at the periapex. Modulating these responses through enhancing anti-inflammatory cellular and molecular pathways and inhibiting pro-inflammatory mediators would favour regeneration and healing over tissue destruction. Therefore, decellularized ECM scaffolds could be promising scaffolds for REPs as they were reported to have an important role in inflammation modulation by triggering anti-inflammatory response that promotes pulp regeneration [7, 98, 99].

Despite the lack of statistical significance between the groups which may be due to the small sample size and short duration of the study, we showed that the BC and i-PRF groups had the highest amount of intracanal calcified tissue while the P-ECM and HA groups had less periapical inflammation. Furthermore, the P-ECM showed the formation of tissues that were more like connective tissue than the other groups highlighting a clear advantage. Furthermore, these newly developed hydrogels can be beneficial as scaffolds in cases where induction of bleeding is difficult. Additionally, they can be available as off-the-shelf readily available products, contrary to platelet-rich concentrates which need to be prepared chairside and eventually require an invasive procedure which may not be feasible for all patients. However, confirmation of the results of the current study requires further optimization of the scaffold and a larger long-term study. In this report, we primarily documented the in vitro preparation and characterization of the material in association with a proof-of-concept demonstration of potential application of this material in vivo.

Nonetheless, aiming to “regenerate” a fully functional dentin/pulp complex in mature permanent teeth may be difficult since embryologic structures, such as Hertwig’s epithelial root sheath and the apical papilla required to ensure biological development of dentin/pulp tissues, have been lost [100]. However, several reports of REPs performed in both immature and mature teeth have successfully demonstrated not only resolution of signs and symptoms and periapical healing but regaining of sensibility as well which indicates the formation of an innervated new vital tissue and the possible survival of stem/progenitor cell populations following long-standing periapical infection [71, 101, 102].

In conclusion, we report the successful preparation and characterization of a decellularized bovine dental pulp-derived extracellular matrix (P-ECM) scaffold in hydrogel form that can be used as a structural and functional biomimetic scaffold for REPs. This tissue specific hydrogel could have additional advantages in REPs such as the cost-effectiveness of the scaffold, its ease of application conforming to irregular canal shapes, and the possibility of storing the scaffold as an off-the-shelf product that can be readily available for faster clinical translation especially in cases where induction of bleeding is difficult and to reduce incidence of intracanal calcification following REPs. Bovine pulp tissue may represent a valuable and abundant material that could be utilized for preparing a significant amount of P-ECM hydrogel. Indeed, in this study, fifteen bovine molars yielded enough pulp tissue that was sufficient to prepare 250–280 ml of P-ECM hydrogel. However, further research is still needed to optimize the concentration of growth factors within the prepared hydrogel and the possibility of cross-linking to other biological materials that can enhance its physical and mechanical properties. Moreover, long-term follow up is necessary in future studies to further evaluate the clinical performance of such scaffolds. With further optimization, the use of such a scaffold could potentially re-establish the native microenvironment for dental pulp regeneration.

## Electronic supplementary material

Below is the link to the electronic supplementary material.

Supplementary Material 1

Supplementary Material 2

Supplementary Material 3

Supplementary Material 4

Supplementary Material 5

## Data Availability

All data generated or analyzed during this study are included in this published article and its additional files.

## Abbreviations

AB: Alcian blue
ARRIVE: The Animal Research: Reporting of In Vivo Experiments
BC: Blood clot
BCA: Bicinchoninic acid
bFGF: Basic fibroblast growth factor
BMP-2: Bone morphogenetic protein-2
CBCT: Cone beam computed tomography
DNA: Deoxyribonucleic acid
dsDNA: Double stranded deoxyribonucleic acid
EDTA: Ethylenediamine tetraacetic acid
ECM: Extracellular matrix
ELISA: Enzyme-linked Immunosorbent Assay
GAGs: Glycosaminoglycans
GIC: Glass ionomer cement
HA: Hyaluronic acid
HCl: Hydrochloric acid
H&E: Hematoxylin and eosin
i-PRF: Injectable platelet rich fibrin
IRM: Intermediate restorative material
MT: Masson’s trichrome
MTA: Mineral trioxide aggregate
MTT assay: 3-(4,5-dimethylthiazol-2-yl)-2,5-diphenyl-2 H-tetrazolium bromide
NaOH: Sodium hydroxide
PBS: Phosphate buffered saline
P-ECM: Pulp extracellular matrix
PRAISE: Preferred Reporting Items for Animal Studies in Endodontology
PVDF: Polyvinylidene fluoride
rDPSCs: Rabbit dental pulp stem cells
REPs: Regenerative endodontic procedures
RMGIC: Resin modified glass ionomer cement
RT: Room temperature
SEM: Scanning electron microscope
TGF𝛽-1: Transforming growth factor beta 1
VEGF: Vascular endothelial growth factor
