# Supplementary material for: Bovine pulp extracellular matrix hydrogel for regenerative endodontic applications: in vitro characterization and in vivo analysis in a necrotic tooth model
Source: Head Face Med. 2024 Oct 22;20:61. doi: 10.1186/s13005-024-00460-y (PMC11494807; doi:10.1186/s13005-024-00460-y)
Supplement: Supplementary file 3 — Supplementary Material 3 [file 13005_2024_460_MOESM3_ESM.docx]

**
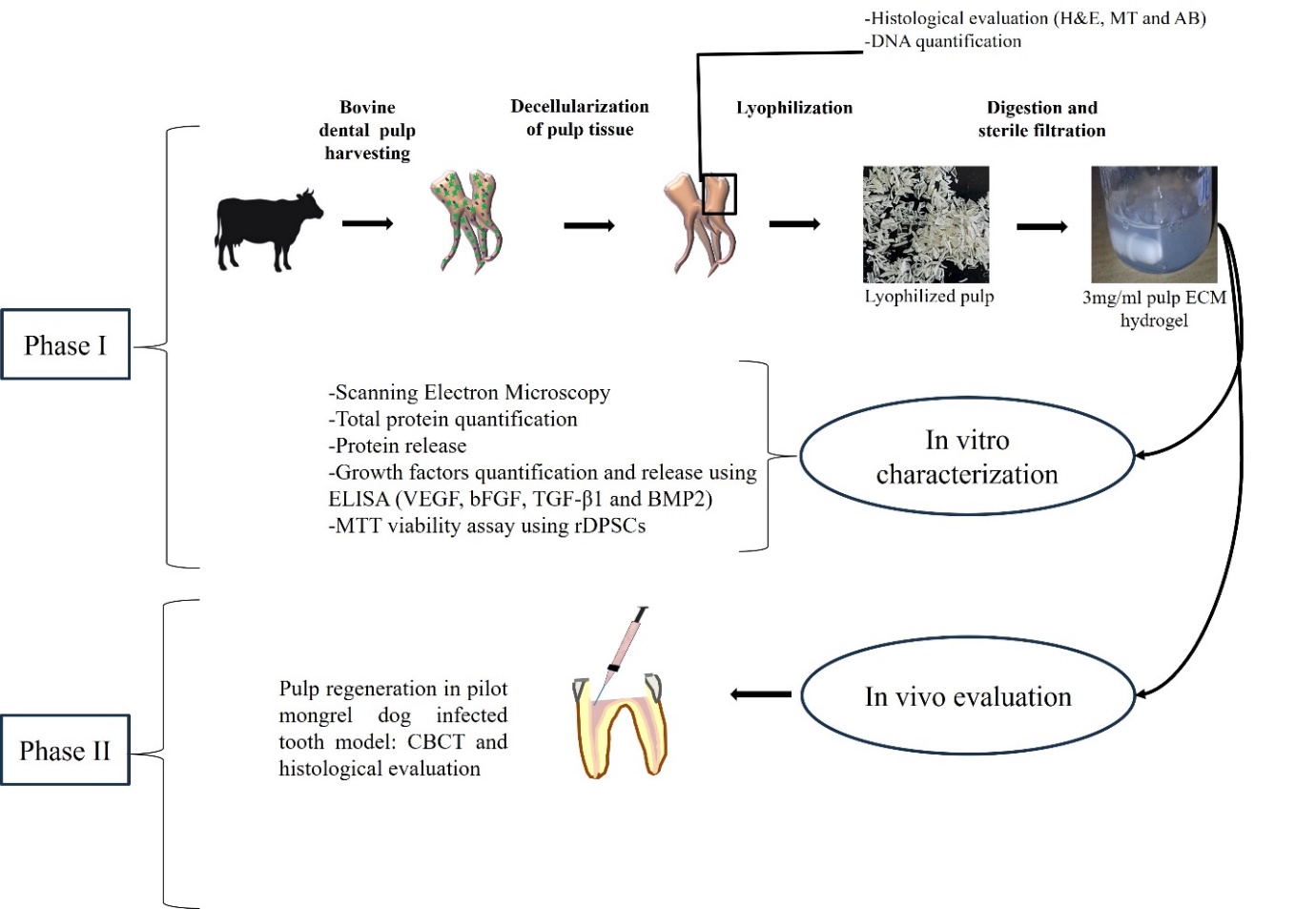
**

**Supplementary figure 1: Scheme of the experimental design illustrating the steps for the decellularization protocol, hydrogel preparation and characterization.** (**AB**, alcian blue stain; **bFGF**, basic fibroblast growth factor; **BMP-2**, Bone morphogenetic protein 2; **CBCT**, cone beam computed tomography; **ELISA**, enzyme-linked immunosorbent assay; **H&E**, hematoxylin and eosin; **MT**, Masson trichrome stain; **MTT**, 3-(4,5-dimethylthiazol-2-yl)-2,5-diphenyl-2H-tetrazolium bromide assay; **rDPSCs**, rabbit dental pulp stem cells; **TGF-β1**, transforming growth factor β; **VEGF**, vascular endothelial growth factor).
