# Supplementary material for: Bovine pulp extracellular matrix hydrogel for regenerative endodontic applications: in vitro characterization and in vivo analysis in a necrotic tooth model
Source: Head Face Med. 2024 Oct 22;20:61. doi: 10.1186/s13005-024-00460-y (PMC11494807; doi:10.1186/s13005-024-00460-y)
Supplement: Supplementary file 4 — Supplementary Material 4 [file 13005_2024_460_MOESM4_ESM.docx]

**Table 1: Comparison of DNA quantification (ng/mg) by nanodrop between decellularized and native pulp**

|  | **Decellularized Pulp** | **Native pulp** |
| --- | --- | --- |
| Mean ±SD | 28.23 ±7.87 | 369.5 ±14.78 |
| 95% CI | 15.70, 40.75 | 236.7, 502.2 |
| Median (Min – Max) | 30.65 (16.80 – 34.80) | 369.5 (359.0 – 379.9) |
| *p* value | <0.0001* | |

*Statistically significant different at *p* value≤0.05

**Table 2:** **Comparison of DNA quantification (ng/mg) by quantifluor dsDNA dye between decellularized and native pulp (ng/mg)**

|  | **Decellularized pulp** | **Native pulp** |
| --- | --- | --- |
| Mean ±SD | 21.18 ±1.75 | 182.4 ±5.19 |
| 95% CI | 19.34, 23.02 | 135.8, 229.1 |
| Median (Min – Max) | 21.41 (18.94 – 23.55) | 182.4 (178.7 – 186.1) |
| *p* value | <0.0001* | |

*Statistically significant different at *p* value≤0.05

**Table 3: Total Protein (µg/ml) in Pulp ECM Hydrogel**

|  | **P-ECM hydrogel** |
| --- | --- |
| Mean ±SD | 439.0 ±123.4 |
| 95% CI | 324.9, 553.2 |
| Median (Min – Max) | 358.1 (308.9 – 595.5) |

**Table 4: Protein release (ug/ml) in pulp ECM hydrogel**

|  |  | **P-ECM hydrogel** |
| --- | --- | --- |
| 0 day | Mean ±SD | 147.56 ±6.29 |
|  | 95% CI | 137.55, 157.57 |
|  | Median (Min - Max) | 147.51 (140.99 – 154.23) |
| 1 day | Mean ±SD | 138.21 ±35.03 |
|  | 95% CI | 82.48, 193.94 |
|  | Median (Min – Max) | 137.46 (107.63 – 170.29) |
| 5 days | Mean ±SD | 114.04 ±30.75 |
|  | 95% CI | 65.12, 162.97 |
|  | Median (Min – Max) | 111.82 (85.96 – 146.58) |
| 14 days | Mean ±SD | 109.79 ±13.12 |
|  | 95% CI | 88.91, 130.66 |
|  | Median (Min – Max) | 115.40 (90.24 – 118.10) |
| *P* value | | 0.058 |

*Statistically significant different at *p* value≤0.05

**Table 5: Release of VEGF (ng/L) in pulp ECM hydrogel**

|  |  | **P-ECM hydrogel** |
| --- | --- | --- |
| 0 day | Mean ±SD | 1839.90 ±68.31 |
|  | 95% CI | 1226.19, 2453.61 |
|  | Median | 1839.90 |
|  | Min - Max | 1791.60 – 1888.20 |
| 1 day | Mean ±SD | 1841.30 ±79.05 |
|  | 95% CI | 1131.02, 2551.58 |
|  | Median | 1841.30 |
|  | Min – Max | 1785.40 – 1897.20 |
| 7 days | Mean ±SD | 1753.00 ±59.40 |
|  | 95% CI | 1219.34, 2286.66 |
|  | Median | 1753.00 |
|  | Min – Max | 1711.00 – 1795.00 |
| ***p* value** | | 0.607 |

*Statistically significant different at *p* value≤0.05

**Table 6: Release of bFGF (ng/L) in pulp ECM hydrogel**

|  |  | **P-ECM hydrogel** |
| --- | --- | --- |
| 0 day | Mean ±SD | 1249.05 ±50.68 |
|  | 95% CI | 793.73, 1704.38 |
|  | Median (Min – Max) | 1249.05 (1213.22 – 1284.89) |
| 1 day | Mean ±SD | 1330.14 ±117.57 |
|  | 95% CI | 273.79, 2386.49 |
|  | Median (Min – Max) | 1330.14 (1247.00 – 1413.28) |
| 5 days | Mean ±SD | 1336.49 ±147.98 |
|  | 95% CI | 6.94, 2666.03 |
|  | Median (Min – Max) | 1336.49 (1231.85 – 1441.13) |
| 14 days | Mean ±SD | 1012.24 ±5.65 |
|  | 95% CI | 961.50, 1062.97 |
|  | Median (Min – Max) | 1012.24 (1008.24 – 1016.23) |
| ***p* value** | | 0.241 |

*Statistically significant different at *p* value≤0.05

**Table 7: Release of TGF-beta 1 (pg/ml) in pulp ECM hydrogel**

|  |  | **P-ECM hydrogel** |
| --- | --- | --- |
| 0 day | Mean ±SD | 152.76 ±20.54 |
|  | 95% CI | 120.07, 185.44 |
|  | Median (Min – Max) | 152.71 (130.77 – 175.69) |
| 1 day | Mean ±SD | 141.79 ±30.90 |
|  | 95% CI | 92.63, 190.96 |
|  | Median (Min – Max) | 149.74 (99.38 – 168.31) |
| 5 days | Mean ±SD | 112.49 ±21.43 |
|  | 95% CI | 78.38, 146.59 |
|  | Median (Min – Max) | 107.02 (93.41 – 142.50) |
| 14 days | Mean ±SD | 104.46 ±35.58 |
|  | 95% CI | 47.84, 161.08 |
|  | Median (Min – Max) | 87.47 (85.11 – 157.80) |
| ***P* value** | | 0.075 |

*Statistically significant different at *p* value≤0.05

**Table 8: Release of BMP2 (pg/ml) in pulp ECM hydrogel**

|  |  | **P-ECM hydrogel** |
| --- | --- | --- |
| 0 day | Mean ±SD | 37.56 ±3.44 |
|  | 95% CI | 6.65, 68.46 |
|  | Median (Min – Max) | 37.56 (35.12 – 39.99) |
| 1 day | Mean ±SD | 49.03 ±8.76 |
|  | 95% CI | -29.65, 127.71 |
|  | Median (Min – Max) | 49.03 (42.84 – 55.22) |
| 5 days | Mean ±SD | 34.30 ±6.39 |
|  | 95% CI | -23.09, 91.69 |
|  | Median (Min – Max) | 34.30 (29.78 – 38.82) |
| 14 days | Mean ±SD | 25.99 ±2.82 |
|  | 95% CI | 0.68, 51.30 |
|  | Median (Min – Max) | 25.99 (23.99 – 27.98) |
| ***p* value** | | 0.145 |

*Statistically significant different at *p* value≤0.05
