# Supplementary material for: Bovine pulp extracellular matrix hydrogel for regenerative endodontic applications: in vitro characterization and in vivo analysis in a necrotic tooth model
Source: Head Face Med. 2024 Oct 22;20:61. doi: 10.1186/s13005-024-00460-y (PMC11494807; doi:10.1186/s13005-024-00460-y)
Supplement: Supplementary file 5 — Supplementary Material 5 [file 13005_2024_460_MOESM5_ESM.docx]

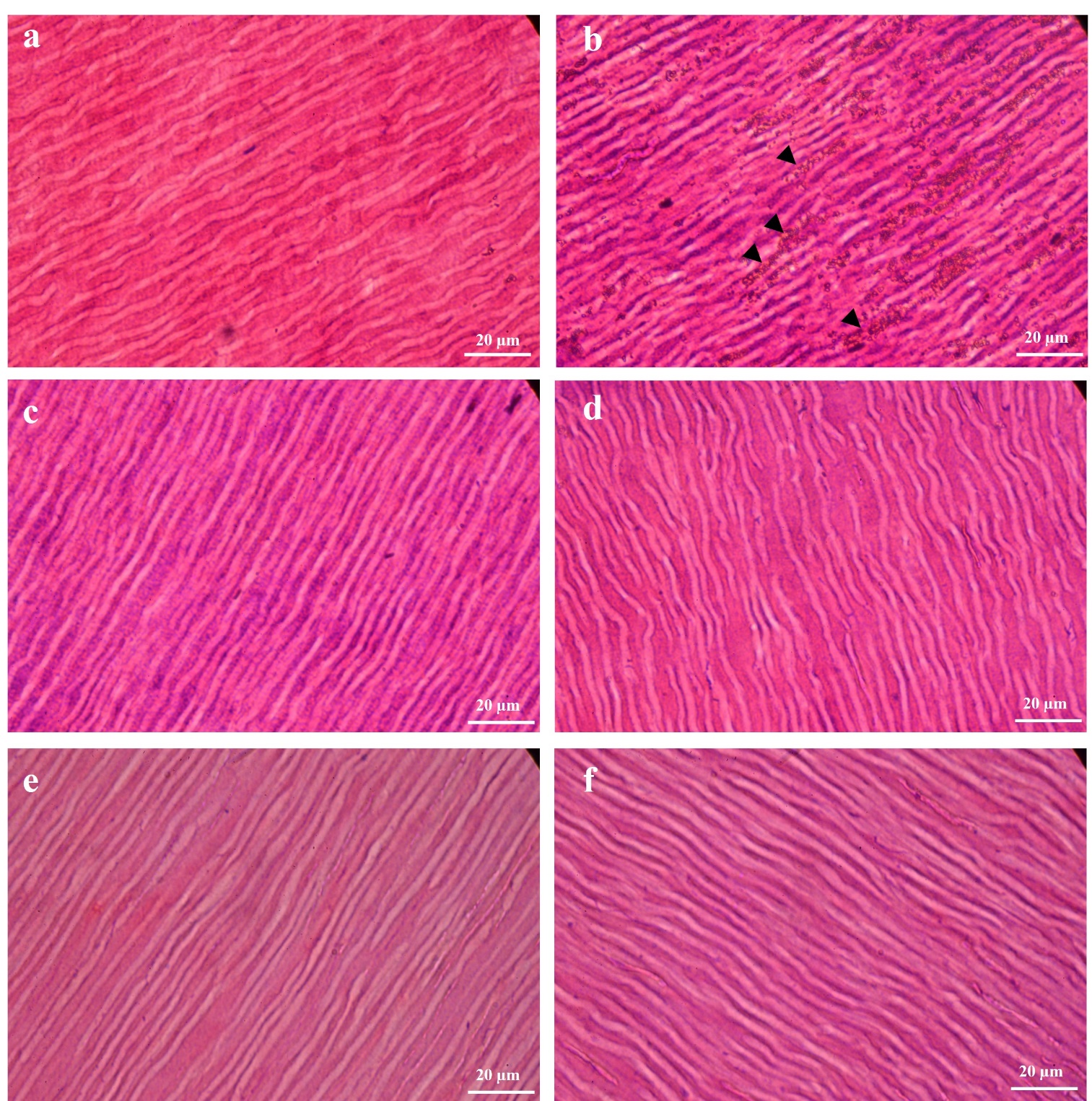


**Supplementary figure 1: Light micrographs showing the dentinal tubules of representative samples for control and experimental groups (a)** Light micrograph showing the dentinal tubules of the positive control sample (intact tooth) free from bacterial cells **(b)** Light micrograph showing the dentinal tubules of the negative control sample (infected tooth) with clusters of bacterial aggregation (black arrow heads) **(c,d,e&f)** Light micrograph showing the dentinal tubules of the BC-treated, i-PRF-treated, HA-treated and P-ECM-treated samples, respectively, with no detectable bacterial cells (Gram stain: a,b,c,d,e&f); (1000x: a,b,c,d,e&f)
