# Supplementary material for: Bovine pulp extracellular matrix hydrogel for regenerative endodontic applications: in vitro characterization and in vivo analysis in a necrotic tooth model
Source: Head Face Med. 2024 Oct 22;20:61. doi: 10.1186/s13005-024-00460-y (PMC11494807; doi:10.1186/s13005-024-00460-y)
Supplement: Supplementary file 5 [file 13005_2024_460_MOESM1_ESM.docx]

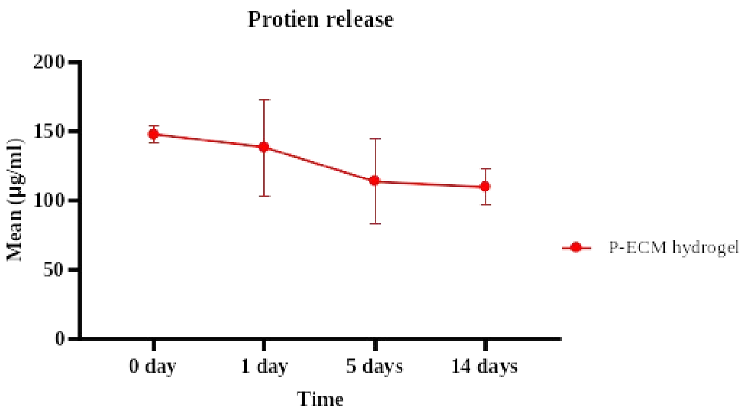
Protien release
200
150
100
50
0
Mean (µg/ml)
0 day
1 day
5 days
14 days
Time
P-ECM hydrogel

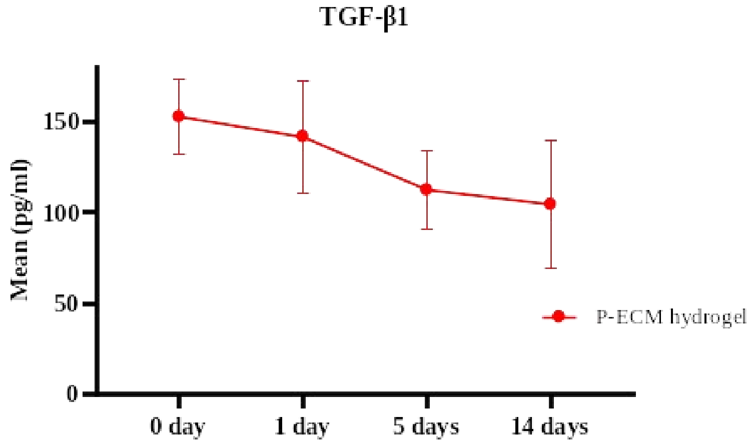
TGF-β1
150
100
50
0
Mean (pg/ml)
0 day
1 day
5 days
14 days
P-ECM hydrogel

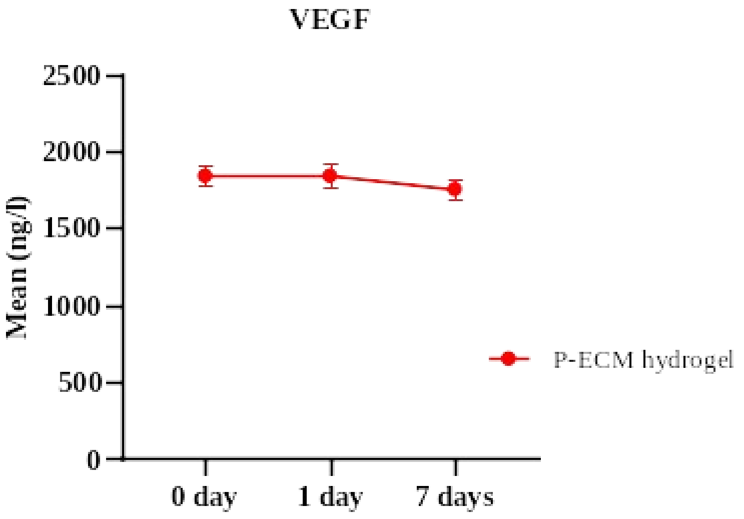
VEGF
2500
2000
1500
1000
500
0
Mean (ng/l)
0 day
1 day
7 days
P-ECM hydrogel

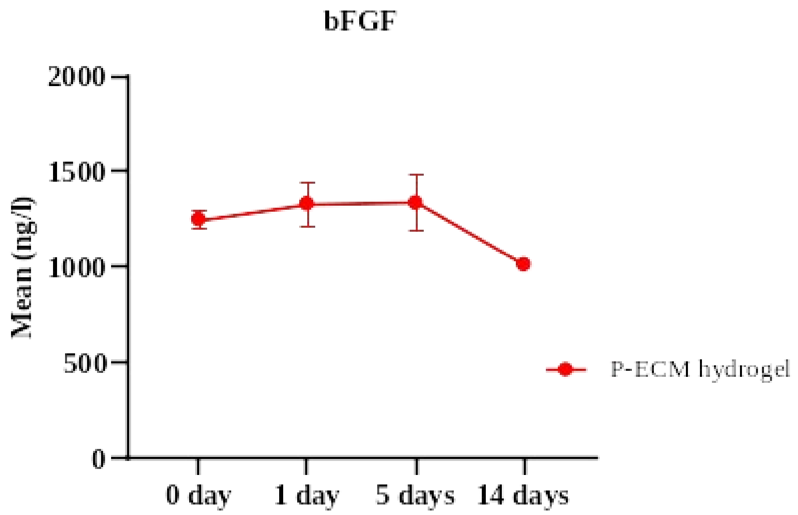
bFGF
Mean (ng/l)
2000
1500
1000
500
0
0 day
1 day
5 days
14 days
P-ECM hydrogel

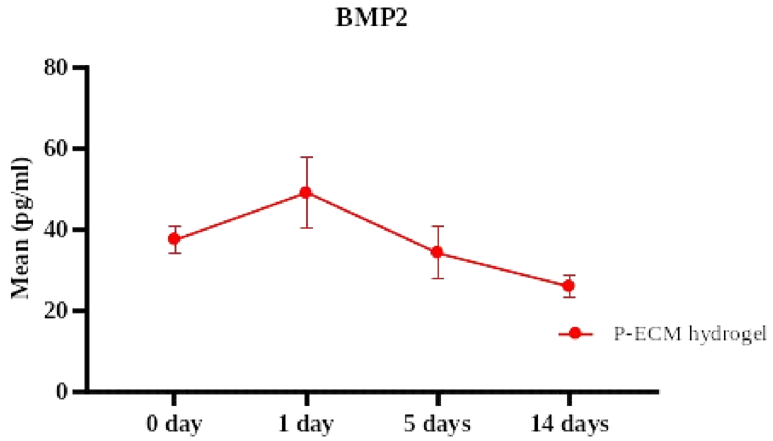
BMP2
Mean (pg/ml)
80
60
40
20
0
0 day
1 day
5 days
14 days
P-ECM hydrogel
